# A Novel Small-Molecule Inhibitor of the *Mycobacterium tuberculosis* Demethylmenaquinone Methyltransferase MenG Is Bactericidal to Both Growing and Nutritionally Deprived Persister Cells

**DOI:** 10.1128/mBio.02022-16

**Published:** 2017-02-14

**Authors:** Paridhi Sukheja, Pradeep Kumar, Nisha Mittal, Shao-Gang Li, Eric Singleton, Riccardo Russo, Alexander L. Perryman, Riju Shrestha, Divya Awasthi, Seema Husain, Patricia Soteropoulos, Roman Brukh, Nancy Connell, Joel S. Freundlich, David Alland

**Affiliations:** a Division of Infectious Disease, Department of Medicine, and the Ruy V. Lourenço Center for the Study of Emerging and Reemerging Pathogens, Rutgers University-New Jersey Medical School, Newark, New Jersey, USA; b Department of Pharmacology, Physiology, and Neuroscience, Rutgers University-New Jersey Medical School, Newark, New Jersey, USA; c Department of Chemistry-Rutgers University-Newark College of Arts and Sciences, Newark, New Jersey, USA; d Genomics Center, Rutgers University-New Jersey Medical School, Newark, New Jersey, USA; e Department of Microbiology, Biochemistry and Molecular Genetics, Rutgers University-New Jersey Medical School, Newark, New Jersey, USA; Sequella, Inc.

## Abstract

Active tuberculosis (TB) and latent *Mycobacterium tuberculosis* infection both require lengthy treatments to achieve durable cures. This problem has partly been attributable to the existence of nonreplicating *M. tuberculosis* “persisters” that are difficult to kill using conventional anti-TB treatments. Compounds that target the respiratory pathway have the potential to kill both replicating and persistent *M. tuberculosis* and shorten TB treatment, as this pathway is essential in both metabolic states. We developed a novel respiratory pathway-specific whole-cell screen to identify new respiration inhibitors. This screen identified the biphenyl amide GSK1733953A (DG70) as a likely respiration inhibitor. DG70 inhibited both clinical drug-susceptible and drug-resistant *M. tuberculosis* strains. Whole-genome sequencing of DG70-resistant colonies identified mutations in *menG* (*rv0558*), which is responsible for the final step in menaquinone biosynthesis and required for respiration. Overexpression of *menG* from wild-type and DG70-resistant isolates increased the DG70 MIC by 4× and 8× to 30×, respectively. Radiolabeling and high-resolution mass spectrometry studies confirmed that DG70 inhibited the final step in menaquinone biosynthesis. DG70 also inhibited oxygen utilization and ATP biosynthesis, which was reversed by external menaquinone supplementation. DG70 was bactericidal in actively replicating cultures and in a nutritionally deprived persistence model. DG70 was synergistic with the first-line TB drugs isoniazid, rifampin, and the respiratory inhibitor bedaquiline. The combination of DG70 and isoniazid completely sterilized cultures in the persistence model by day 10. These results suggest that MenG is a good therapeutic target and that compounds targeting MenG along with standard TB therapy have the potential to shorten TB treatment duration.

## IMPORTANCE

This study shows that MenG, which is responsible for the last enzymatic step in menaquinone biosynthesis, may be a good drug target for improving TB treatments. We describe the first small-molecule inhibitor (DG70) of *Mycobacterium tuberculosis* MenG and show that DG70 has characteristics that are highly desirable for a new antitubercular agent, including bactericidality against both actively growing and nonreplicating mycobacteria and synergy with several first-line drugs that are currently used to treat TB.

## INTRODUCTION

Treatment of *Mycobacterium tuberculosis* has become increasingly challenging with the rise of drug-resistant strains. Existing treatment of drug-susceptible tuberculosis (TB) requires administration of multiple drugs for a minimum of 6 months; treatment of drug-resistant TB can extend to years and is often unsuccessful (1, 2). TB in humans probably includes several sub-populations of *M. tuberculosis* with differing metabolic states (3, 4). Some populations, for example, those that reside in well-aerated cavities, replicate quickly and are easily killed by a number of drugs, while other populations composed of nonreplicating persistent bacteria are much less drug susceptible (3, 5, 6). Persistent *M. tuberculosis* downregulates metabolic processes required for active growth such as cell wall biosynthesis, and these organisms become relatively or absolutely tolerant to drugs (5, 7–10). New anti-TB drugs with the ability to kill *M. tuberculosis* in both replicating and nonreplicating states could be used to treat drug-resistant TB and also potentially to reduce the required duration of treatment. The effectiveness of TB treatment could be further enhanced if new drugs were synergistic with current therapies (11).

Unlike many bacteria, *M. tuberculosis* cannot support its energy needs through substrate-level phosphorylation. Instead, both actively growing and nonreplicating persistent *M. tuberculosis* bacteria are dependent on respiration to synthesize adequate amounts of ATP (12). The *M. tuberculosis* respiratory chain consists of various electron donors that transfer two electrons to lipoquinone with the help of corresponding dehydrogenases. Lipoquinone then transfers these electrons to the oxidoreductases, which then reduce terminal electron acceptors (13). The electrochemical gradient generated in the process is utilized to synthesize ATP by F_o_F_1_ ATP synthase (4). *M. tuberculosis* has the capacity to utilize numerous electron donors and acceptors depending on its microenvironment (13). However, menaquinone (MK9) and its saturated form [MK-9(II-H_2_)] are the only lipoquinones in *M. tuberculosis* that transfer electrons from dehydrogenases to the terminal electron oxidases (14, 15). Thus, menaquinone represents an essential vulnerable point in the electron transport chain and a prime target for development of new drugs. The absence of menaquinone biosynthesis in humans further supports the druggability of enzymes involved in this pathway. Menaquinone biosynthesis begins with chorismate derived from the shikimate pathway. The conversion of chorismate to isochorismate is the first committed step in menaquinone biosynthesis (16). Isochorismate is then converted to demethylmenaquinone by a cascade of at least 8 different enzymes (15). As the final step in menaquinone biosynthesis, MenG (Rv0558) catalyzes methylation of demethylmenaquinone (DMK9) using *S*-adenosylmethionine (SAM), resulting in formation of menaquinone (12).

Here, we searched for specific inhibitors of respiration in *M. tuberculosis* utilizing a novel respiratory pathway-specific whole-cell-based screen to test a library of known anti-TB compounds provided by GlaxoSmithKline (GSK) (17). We report a novel anti-TB chemotype, GSK1733953A (here renamed DG70), which was discovered through this screen; show that DG70 acts through inhibition of MenG; and investigate the effects and potential therapeutic utility of MenG inhibition.

## RESULTS

### Identification of a biphenyl amide inhibitor from a compound library.

**FIG 1:**
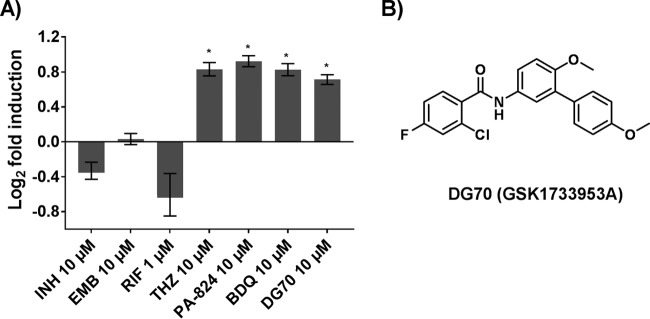
PcydAB induction identifies DG70 as a candidate respiratory inhibitor. (A) The relative induction of the PcydAB::*mWasabi* reporter as measured by determining the relative fluorescence units of each drug-treated culture compared to the DMSO-only control is shown for several anti-TB drugs as well as DG70. Each was tested in triplicate in a 96-well format. Isoniazid (INH), rifampin (RIF), and ethambutol (EMB) were used as negative controls; thioridazine (THZ), bedaquiline (BDQ), and PA824 were used as positive controls (21–23). Means and standard deviations from three independent experiments are shown. DG70 consistently induced Pcyd 1.7-fold over DMSO. The Student *t* test was used to determine statistical significance between DMSO and DG70. *, *P* ≤ 0.05. (B) Molecular structure of DG70.

To identify novel inhibitors of mycobacterial respiration, we developed a whole-cell respiratory pathway-specific screen that combined the advantages of a whole-cell screen with those of a target-based approach (18). A *Mycobacterium bovis* BCG (BCG) reporter strain was created by fusing the putative promoter (PcydAB) for the *M. tuberculosis cydAB* operon to an *mWasabi* reporter. The *cydAB* operon has been shown to be upregulated in response to respiratory poisoning along with low oxygen tension during the transition to anaerobiosis in *M. tuberculosis* (19, 20). We used this reporter strain to detect possible respiratory inhibitors that are also able to penetrate into live mycobacterial cells. This reporter strain was first validated by testing it against known respiratory inhibitors such as the NADH/succinate dehydrogenase inhibitor thioridazine (THZ), the ATP synthase inhibitor bedaquiline (BDQ), and PA824, which appears to have multiple targets, including the *M. tuberculosis* cytochrome *bc*_1_ and cytochrome *bd* oxidase (21–23). Negative controls included drugs that inhibit or kill *M. tuberculosis* by unrelated mechanisms. Significant induction of the PcydAB-fused *mWasabi* reporter was seen only in cells treated with the respiratory inhibitors (Fig. 1A). We then screened a diverse library of 168 compounds already known to inhibit *M. tuberculosis* but where the mechanism of action was unknown (17). DG70, a biphenyl benzamide (Fig. 1B), was selected from a number of positive screening hits because it consistently induced the PcydAB promoter at least 1.7-fold over the dimethyl sulfoxide (DMSO) control (*P* < 0.05) (Fig. 1A). The induction was comparable to those with THZ, BDQ, and PA824 (21–23) (Fig. 1A) and showed a peak of 3.1-fold induction when a full range of DG70 concentrations were tested (see Fig. S1 in the supplemental material).

10.1128/mBio.02022-16.3 FIG S1  PcydAB induction dose response for DG70. PcydAB fold induction over vehicle dose-response profile of BCG reporter strain treated with 2-fold serial dilutions of DG70 in a fluorescence assay at a maximum of 8× MIC in a 96-well plate format. Isoniazid (INH) was used as the negative control, bedaquiline (BDQ) was used as the positive control, and DMSO was the vehicle used to dissolve compound. Download FIG S1, DOCX file, 0.1 MB. Copyright © 2017 Sukheja et al. 2017 Sukheja et al. This content is distributed under the terms of the Creative Commons Attribution 4.0 International license.

### DG70 is potent against *M. tuberculosis* complex.

**FIG 2:**
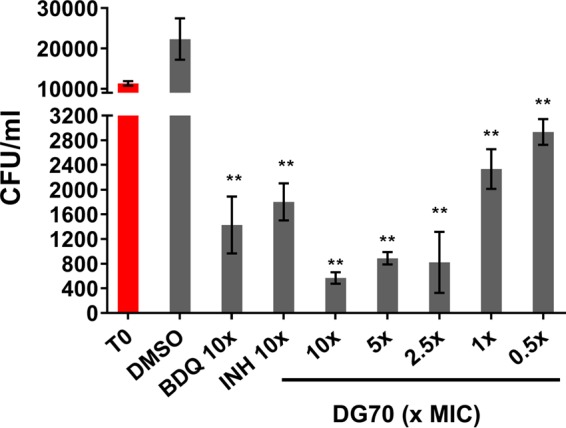
Dose response of DG70 against *M. tuberculosis* mc^2^6206 in J774A.1 murine macrophages. J774A.1 macrophages were infected with 10^4^ CFU/ml of *M. tuberculosis* (T0, red bar) and treated with DG70 at final concentrations of 0.5, 1, 2.5, 5, and 10× its MIC. Macrophages treated with either bedaquiline (BDQ) or isoniazid (INH) at 10× MIC were used as positive controls. CFU were determined after 3 days of treatment and were back calculated to CFU per milliliter. T0 shows the initial CFU used for infecting macrophages. Means and standard deviations from three independent experiments are shown. The Student *t* test was used to determine statistical significance between DMSO and different treatments. **, *P* ≤ 0.01.

We tested the activity of DG70 against a panel of clinical and laboratory drug-susceptible and drug-resistant *M. tuberculosis* strains. DG70 had a MIC of 4.8 µg/ml against *M. tuberculosis* H37Rv and 1.2 to 9.6 µg/ml against drug-resistant strains, suggesting a novel molecular target (Table 1). The MIC variation in clinical strains was similar to that observed among clinical drug-susceptible strains when tested with isoniazid (INH) and rifampin (RIF) (24). DG70 lacked activity against nontuberculous mycobacteria (NTM) and a panel of Gram-positive, Gram-negative, ESKAPE (*Enterococcus faecium*, *Staphylococcus aureus*, *Klebsiella pneumoniae*, *Acinetobacter baumannii*, *Pseudomonas aeruginosa*, and *Enterobacter* species), and surrogate select agent bacteria (Table S1). Consistent with these findings, an alignment of MenG amino acid sequences showed that NTM had only 64.5 to 88% identity to *M. tuberculosis* (Fig. S2), and molecular docking studies of DG70 in its predicted MenG binding sites identified significant steric clashes in the NTM MenG sequences studied (Text S1). Of the other bacteria tested, none of their MenG sequences had more than 40.6% amino acid identity to *M. tuberculosis* MenG. Cytotoxicity testing in Vero cells showed a 50% cytotoxic concentration (CC_50_) of >77 µg/ml and a selectivity index (SI = CC_50_/MIC for *M. tuberculosis*) of >30. DG70 also inhibited intracellular *M. tuberculosis* growth in J774A.1 macrophages at concentrations equivalent to its *in vitro* MIC (Fig. 2).

10.1128/mBio.02022-16.1 TEXT S1  Description of detailed chemical synthesis and computational modeling, docking methods, and supplemental results. Download TEXT S1, DOCX file, 0.1 MB. Copyright © 2017 Sukheja et al. 2017 Sukheja et al. This content is distributed under the terms of the Creative Commons Attribution 4.0 International license.

10.1128/mBio.02022-16.4 FIG S2  Multiple sequence alignment of *M. tuberculosis* MenG versus other mycobacteria against which the activity of DG70 was tested. A multiple sequence alignment of MenG from *M. tuberculosis*, BCG (*M. bovis*), *M. marinum*, *M. avium*, *M. smegmatis*, *M. fortuitum*, and *M. abscessus* was performed in Discovery Studio 4.5. Color legend: green, identical residues; cyan, strong conservation; magenta, weak conservation; no highlight, nonmatching. The consensus alignment was determined using a 51% sequence identity cutoff. Download FIG S2, DOCX file, 1.2 MB. Copyright © 2017 Sukheja et al. 2017 Sukheja et al. This content is distributed under the terms of the Creative Commons Attribution 4.0 International license.

10.1128/mBio.02022-16.8 TABLE S1  DG70 MICs in nontuberculosis mycobacterial strains, ESKAPE pathogens, and surrogate select agents. Download TABLE S1, DOCX file, 0.01 MB. Copyright © 2017 Sukheja et al. 2017 Sukheja et al. This content is distributed under the terms of the Creative Commons Attribution 4.0 International license.

**TABLE 1:** DG70 MICs in drug-susceptible, drug-resistant, and *menG*-overexpressing strains of *M. tuberculosis* and BCG^a^

Strain and supplement	Drug resistance	Strain type	MIC_90_ (µg/ml) of drug:	
			DG70	INH
*M. tuberculosis*				
H37Rv	No	Lab	4.8	0.05
H37Rv + 400 µM MK4	No	Lab	9.6	0.05
210	No	Clinical	4.8	0.05
692	No	Clinical	1.2	0.05
TDR 31	INH, RIF, EMB, KAN, SM, CAP	Clinical	1.2	>3.15
TDR 116	INH, EMB, PAS	Clinical	9.6	>3.15
TDR 36	INH, RIF, EMB	Clinical	3.13	>3.15
TDR 91	RIF, EMB, INH	Clinical	4.8	>3.15
BDQ^r^ H37Rv	BDQ	Clinical	4.8	0.05
PA824^r^ H37Rv	PA824	Lab	4.8	0.05
				
*M. bovis*				
BCG	No	Lab	2.4	0.05
BCG + 300 µM MK4	ND	Lab	9.8	ND
BCG::pMV306PHsp60	HYG	Lab	2.4	ND
BCG::pMV306PHsp60-*menG_*Wt	HYG	Lab	9.8	ND
BCG::pMV306PHsp60-*menG_*F118L	HYG	Lab	>40	ND
BCG::pMV306PHsp60-*menG_*V20A	HYG	Lab	20	ND

a Abbreviations: EMB, ethambutol; RIF, rifampin; INH, isoniazid; KAN, kanamycin; SM, streptomycin; CAP, capreomycin; PAS, *para*-aminosalicylic acid; BDQ, bedaquiline; HYG, hygromycin B; ND, not determined. MIC_90_ values were determined by microtiter alamarBlue assay. All MIC_90_ assays were performed in triplicates.

### DG70 targets MenG in *M. tuberculosis*.

**FIG 3:**
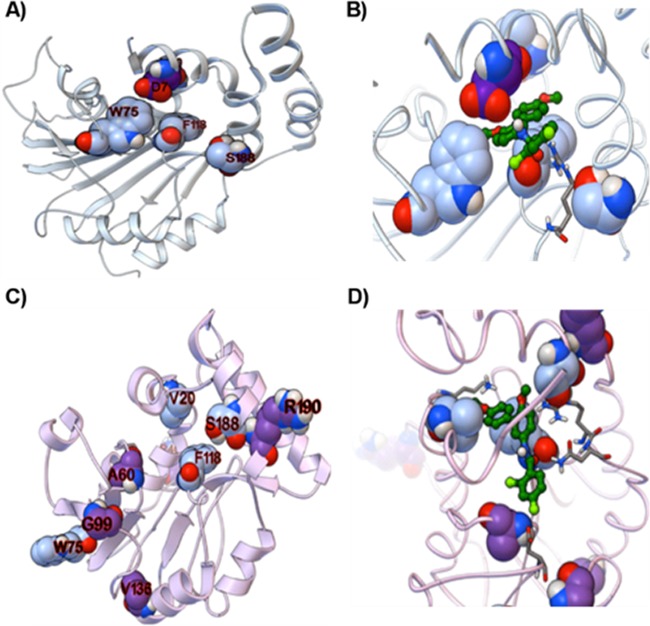
Predicted binding modes of DG70 with models of MenG. (A and B) Multitemplate-based homology model of MenG from MODELLER. (C and D) Model made by threading MenG onto the known class, architecture, topology, and homology domains in PsiPRED. (A and D) Ribbon representation of these two MenG models. The residues that mutate in *M. tuberculosis* upon DG70 treatment are shown according to the Corey, Pauling, and Koltun scheme with blue carbon atoms, and residues that mutate in BCG are displayed with purple carbon atoms. Two representative docked modes of DG70 are presented in panels B and D as ball-and-stick models with dark green carbon atoms.

**FIG 4:**
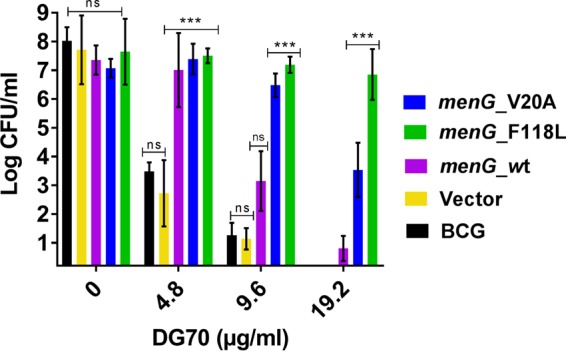
Growth inhibition of mycobacteria overexpressing wild-type and mutant *menG*. BCG strains constitutively overexpressing wild-type* M. tuberculosis menG* and DG70-resistant* M. tuberculosis menG* mutants containing either F118L or V20A substitutions were incubated with DG70 at the indicated concentrations for 7 days, after which each culture was plated and CFU were counted after 4 weeks of incubation at 37°C. CFU counts were back calculated to CFU per milliliter. Means and standard deviations from three independent experiments are shown. The Student *t* test was used to determine statistical significance between DMSO and different treatments. ***, *P* ≤ 0.01; ns, no significant difference.

We searched for the target of DG70 by selecting for DG70-resistant mutants in *M. tuberculosis* and BCG. Two *M. tuberculosis* isolates (70P3 and 70P7) that were 8- and >32-fold resistant to DG70, respectively, were whole genome sequenced. Each mutant had a unique single nucleotide polymorphism (SNP) in *menG* (*rv0558*), leading to amino acid substitutions F118L and V20A, respectively (Table 2). Targeted sequencing of *menG* in additional DG70-resistant *M. tuberculosis* and BCG mutants revealed other *menG* SNPs (Table 2). Moreover, docking results against homology models of MenG predicted that DG70 would interact favorably with the four residues mutated in DG70-resistant *M. tuberculosis* isolates. DG70 interacted with two to three of the residues mutated in DG70-resistant *M. tuberculosis* in each top-ranked docked mode (Fig. 3A and B), and all four residues interacted with DG70 in the entire collection of top-ranked modes (Fig. 3C and D). Detailed descriptions of the plausible binding modes and the sequence variability of these key positions are provided in Text S1. To further investigate whether MenG was targeted by DG70, we used the *hsp60* promoter to overexpress *menG* in BCG on an integrative plasmid, overexpressing either wild-type *menG* or the DG70-resistant F118L (MenG-F118L) or V20A (MenG-V20A) *menG* mutants (Fig. 4). Compared to the strain containing empty vector, overexpressing wild-type *menG* conferred a moderate degree of DG70 resistance and overexpressing mutant *menG* conferred increased levels of resistance (Table 1). In fact, the strain overexpressing *menG*_F118L was completely resistant to even 20 µg/ml of DG70. These data strongly suggested that DG70 targets MenG in *M. tuberculosis*. Additionally, MenG has been shown to be essential in *M. tuberculosis* by Himar1-based transposon mutagenesis (25) and thus is a plausible drug target.

**TABLE 2:** Single nucleotide polymorphisms detected in *M. tuberculosis* and *M. bovis* BCG spontaneous mutants resistant to DG70

Strain	DG70 MIC_90_ (µg/ml)	Gene(s) mutated	Amino acid change
H37Rv			
70P3^a^	>80	*menG*, *sigL*	V20A
70P7^a^	40	*menG*, *ponA1*	F118L
DRM1	>80	*menG*	W75A
DRM7	>80	*menG*	S188A
			
BCG			
70B1	19.2	*menG*	A60V
70B2	19.2	*menG*	A60V
70B4	19.2	*menG*	D7H
70R1	9.6	*menG*	G99V
70R2	9.6	*menG*	G99V
70R5	19.2	*menG*	D7H
70S1	>40	*menG*	R43W
70S3	9.6	*menG*	V136F
70S4	9.6	*menG*	R190M

a Whole-genome-sequenced strains.

### DG70 inhibits menaquinone biosynthesis.

**FIG 5:**
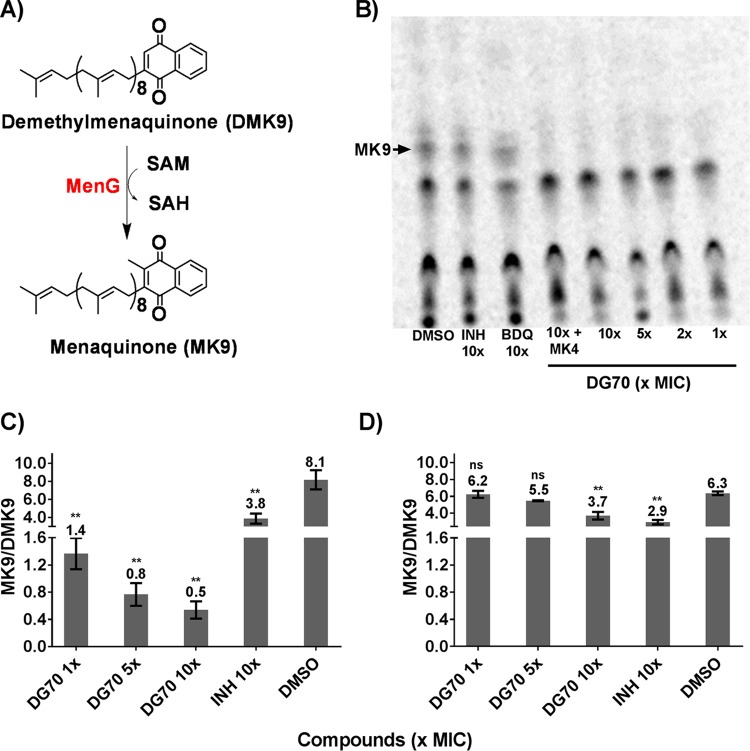
Effect of DG70 on menaquinone biosynthesis. (A) Schematic showing conversion of demethylmenaquinone (DMK9) to menaquinone (MK9) by MenG. SAH, *S*-adenosyl-l-homocysteine. (B) TLC analysis of neutral lipids isolated from BCG labeled with l-[*methyl*-^14^C]methionine after DG70, isoniazid (INH), or bedaquiline (BDQ) treatment. Menaquinone [MK-9(II-H_2_)] was identified by comigration with an authentic standard as observed under UV light. (C and D) Ratios of MK9/DMK9 present in neutral lipid extracts of wild-type BCG (C) and the DG70-resistant BCG mutant (BCG 70B1 MenG-A60V) (D) cultures treated with the indicated compounds as detected by high-resolution mass spectrometry. Means and standard deviations from three independent experiments are shown. The Student *t* test was used to determine statistical significance between DMSO and different treatments. **, *P* ≤ 0.01; ns, no significant difference.

MenG is a demethylmenaquinone methyltransferase that catalyzes one of the last steps of the menaquinone (MK9) biosynthesis pathway by methylation of demethylmenaquinone (15, 25, 26). Consistent with the known function of MenG, we observed that supplementing DG70-treated cultures with 300 to 400 µM of the MK9 analog MK4 partially rescued DG70-treated cells (Table 1). To further investigate the effect of DG70 on menaquinone biosynthesis in whole cells, metabolic labeling was performed using l-[*methyl*-^14^C]methionine (16). *De novo* synthesis of menaquinone in *M. tuberculosis* was monitored by preparative thin-layer chromatography (TLC). Menaquinone [MK-9(II-H_2_)] was identified by comigration with an authentic MK9 standard. Cells treated with as little as 1× MIC of DG70 showed complete suppression of [^14^C]methyl incorporation into menaquinone while the untreated lane as well as INH- or BDQ-treated lanes showed no such effect, strongly suggesting that DG70 was specially inhibiting MenG (Fig. 5A and B). To confirm that the disappearing radiolabeled moiety was in fact menaquinone, an unlabeled fraction of neutral lipids from an untreated BCG culture was also chromatographed on the same TLC. We then eluted the unlabeled material with *R*_*f*_ corresponding to the radioactively labeled material of interest from the silica gel TLC plate and subjected it to high-resolution mass spectrometry (HRMS) with atmospheric pressure chemical ionization (APCI) as described previously (16). The resulting mass spectrum clearly showed the presence of a molecular ion peak with an *m/z* value of 787.6387 atomic mass units (amu), consistent with the predominant mycobacterial menaquinone [MK-9(II-H_2_)]. The fully unsaturated and less abundant form of menaquinone (MK9) with a peak at an *m/z* value of 785.6263 amu was also observed in the corresponding mass spectra. Even though BCG can be partially rescued from DG70 by the addition of 400 µM MK4, in the l-[*methyl*-^14^C]methionine incorporation assay (Fig. 5B, lane 4), external supplementation of MK4 did not circumvent inhibition of [^14^C]methyl incorporation in menaquinone. This suggested that although MK4 can physically substitute for MK9 and maintain the viability of mycobacteria treated with DG70, MK4 does not prevent inhibition of MK9 synthesis by DG70. To conclusively show that DG70 specifically inhibits MenG, we treated BCG with DG70 at 1×, 5×, and 10× MIC in liquid medium for 3 days and then analyzed its neutral lipids through HRMS with APCI. The relative ratio of menaquinone [MK-9(II-H_2_)] (*m/z* 787.6387 amu) to demethylmenaquinone (DMK9) (*m/z* 771.8202 amu) was at least 7-fold lower in DG70-treated cells at 1× MIC than in untreated cells (Fig. 5C). In contrast, a decrease in the ratio of MK-9(II-H_2_)/DMK9 was not observed when strain 70B1, a DG70-resistant BCG mutant, was treated with DG70 at up to 5× MIC of the parent strain. Finally, the ratio of MK-9(II-H_2_)/DMK9 did decrease in strain 70B1 when it was treated with DG70 above its MIC, at 10× MIC of the parent strain (Fig. 5D).

### DG70 inhibits oxygen consumption and ATP synthesis.

**FIG 6:**
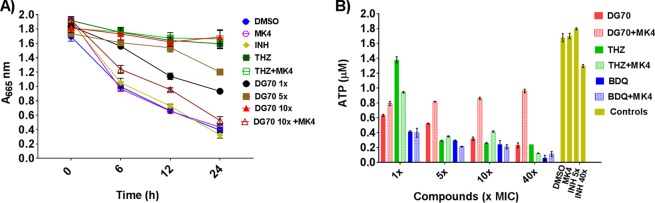
DG70 inhibits oxygen consumption and ATP synthesis in mycobacteria. (A) Effect of DG70 on oxygen consumption. Mycobacterial cells containing 0.01% methylene blue were treated with thioridazine (THZ), isoniazid (INH), or DG70 at the indicated concentrations (×MIC). Cultures were supplemented with MK4 (400 µM) where indicated. Decolorization of methylene blue, as an indicator of oxygen consumption, was monitored at 665 nm. (B) The ATP levels of 1 ml of cellular lysate of BCG (OD_595_ = 0.4) after 3 days of treatment with the indicated compounds are shown. MK4 (400 µM) was added as a supplement as indicated.

Menaquinones are essential electron carriers in the respiratory chain of *M. tuberculosis*, and inhibitors of their biosynthesis would be expected to indirectly affect respiratory ATP synthesis and oxygen utilization. The effect on mycobacterial oxygen consumption was determined by monitoring methylene blue decolorization at 665 nm for 24 h in Parafilm-sealed culture tubes in the presence or absence of DG70. Decolorization of methylene blue indicates utilization of oxygen and a functional respiratory chain in bacteria (19). DG70 strongly prevented decolorization of methylene blue (Fig. 6A). Inhibition of oxygen consumption was comparable to that with the positive THZ-treated control, whereas INH, which does not inhibit respiration, as well as other negative controls, clearly decolorized methylene blue. Intracellular ATP was measured in BCG treated with DG70 or not, and a significant decrease in ATP levels was observed in DG70-treated cells compared to controls (Fig. 6B). Supplementation of the culture medium with 400 µM MK4 rescued the BCG cells from the effects of DG70 on oxygen consumption as well as ATP synthesis (Fig. 6A and B). However, external MK4 (400 µM) supplementation of cultures treated with THZ or BDQ failed to reverse the effect on oxygen consumption and ATP synthesis.

### DG70 is bactericidal against nutrient-deficient, persistent mycobacteria.

**FIG 7:**
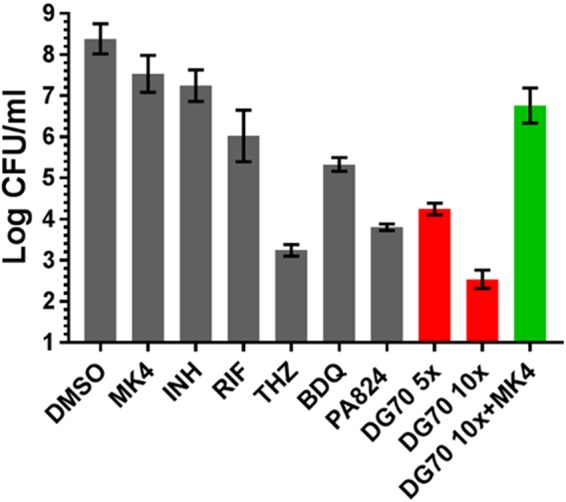
Activity of DG70 against nonreplicating *M. tuberculosis*. *M. tuberculosis* starved for 6 weeks in PBS (nutritionally deprived persistence model) was exposed to various concentration of DG70 and 10× MIC of isoniazid (INH), bedaquiline (BDQ), PA824, thioridazine (THZ), and rifampin (RIF) for 7 days and then plated to determine viability. Menaquinone (MK4) at a 400 µM concentration was used to test for reversal of the killing mediated by DG70 in the nutrient-deprived *M. tuberculosis*. Experiments were carried out in duplicate, and each dilution was plated in triplicate. CFU counts were back calculated to CFU per milliliter. Results are shown as means and standard deviations.

Under nutrient-deficient conditions, mycobacteria enter a nonreplicating state and have been previously shown to become tolerant to most drugs (27). However, respiratory ATP synthesis is essential to both actively growing and persistent mycobacteria (13). Furthermore, menaquinone levels have been shown to decrease in hypoxic cultures of *M. tuberculosis*, and MenA (the enzyme upstream of MenG in menaquinone biosynthesis) inhibition in BCG cultured under nutrient-deficient conditions leads to a 3-log_10_ decrease in CFU (16, 28). These prior results suggested that nonreplicating mycobacteria might be particularly susceptible to MenG inhibition. We therefore tested the activity of DG70 on *M. tuberculosis* persisters using nutrient-deprived culture conditions as an *in vitro* model of the dormancy phenotype. In this model, DG70 resulted in 4-log_10_ and 6.5-log_10_ reductions of CFU after 7 days of treatment at 5× and 10× MIC, respectively (Fig. 7). This activity was far superior to those of all the other known anti-TB drugs tested. The activity of DG70 against a nonreplicating population of *M. tuberculosis* was also better than previously reported for other menaquinone biosynthesis inhibitors (16, 28, 29). Confirming the role of menaquinone inhibition in this model, the effect of DG70 was reversed by 400 µM MK4.

### DG70 interactions with known anti-TB compounds.

Single-drug TB treatment rapidly leads to resistance, and therefore, combination therapies are utilized (30). We performed synergy studies between DG70 and the first-line drugs INH, ethambutol (EMB), and RIF as well as known respiratory inhibitors BDQ, THZ, and PA824 to examine the *in vitro* effect of combined TB treatment. A checkerboard titration in microtiter plates was used as previously described (31). We observed strong synergy between DG70 and INH as well as between DG70 and RIF with ∑FIC (fractional inhibitory concentrations) of 0.4 and 0.5, respectively (Table 3; also see Fig. S4 in the supplemental material). We also examined the effect of treating cultures with DG70 and other respiratory inhibitors (32). The combination of BDQ and DG70 showed promising synergy with a ∑FIC of 0.5. The interactions of DG70 with THZ and PA824 were additive with ∑FIC values of 0.7 and 1.0, respectively.

10.1128/mBio.02022-16.5 FIG S3  Additional predicted binding modes of DG70 with models of MenG. In all panels, the residues that mutate in *M. tuberculosis* upon DG70 treatment are shown as CPK with light blue carbon atoms. Residues that mutate in BCG are displayed with purple carbon atoms, and additional residues that formed strong favorable interactions with the docked mode (but that did not mutate in selection experiments) are shown with gray carbons (either as thin sticks or as CPK). In panels A and B, the multitemplate-based homology model of MenG from MODELLER that had the top DOPE score is displayed, with panel B displaying the docked binding mode of DG70 to this model, using the ball-and-stick model with green carbon atoms. Panels C and D depict the multitemplate-based homology model from MODELLER that had the second highest DOPE score; the docked mode of DG70 is displayed in panel D (with Arg121 shown as thin gray sticks). Panels E and F show the model made by threading MenG onto the known CATH domains using pDomTHREADER in PsiPRED, and each of these panels displays a different top-ranked docked mode that was produced against this target. Panels G to I display the MenG model created by threading its sequence onto the crystal structure of PDB identifier 4obx using pGenTHREADER in PsiPRED, and two different views of the same docked mode are depicted in panels H and I. Download FIG S3, DOCX file, 2.8 MB. Copyright © 2017 Sukheja et al. 2017 Sukheja et al. This content is distributed under the terms of the Creative Commons Attribution 4.0 International license.

10.1128/mBio.02022-16.6 FIG S4  Representative results from the alamarBlue checkerboard assay for DG70 and known anti-TB drugs in *M. tuberculosis*. *M. tuberculosis* H37Rv was treated with 2-fold serial dilutions of DG70 and known anti-TB drugs isoniazid (INH) (A), rifampin (RIF) (B), ethambutol (EMB) (C), and bedaquiline (BDQ) (D). The viability is determined by resazurin (blue), which is converted to pink (resorufin) by viable bacteria. Download FIG S4, DOCX file, 0.8 MB. Copyright © 2017 Sukheja et al. 2017 Sukheja et al. This content is distributed under the terms of the Creative Commons Attribution 4.0 International license.

**TABLE 3:** Synergy between DG70 and antitubercular drugs

Drug combination	MIC (µM)		FIC	FIC index, category^a^
	Alone	In combination		
BDQ	0.39	0.16	0.4	0.5, synergistic
DG70	12.5	1.5	0.1	
				
PA824	0.1	0.1	1.0	1.0, additive
DG70	12.5	0.35	0.03	
				
THZ	6.25	3.12	0.5	0.7, additive
DG70	12.5	3.12	0.2	
				
INH	0.39	0.09	0.2	0.4, synergistic
DG70	12.5	3.12	0.2	
				
RIF	0.02	0.005	0.25	0.5, synergistic
DG70	12.5	3.1	0.25	
				
EMB	6.25	3.12	0.5	0.6, additive
DG70	12.5	1.5	0.12	

a FIC categories: ≤0.5, synergistic; >0.5 to ≤1, additive; >1 to <4, no interaction; ≥4, antagonism.

### Enhanced killing of BCG with DG70 in combination with BDQ, INH, or PA824.

**FIG 8:**
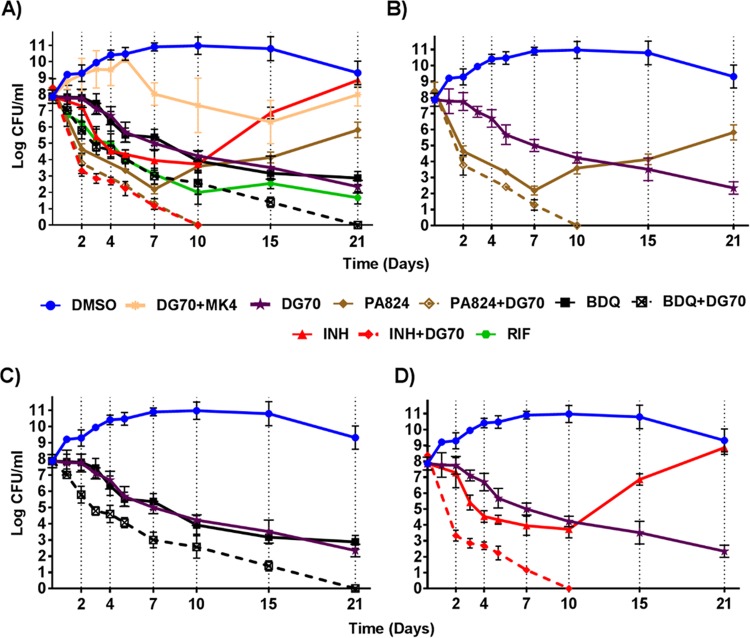
*In vitro* bactericidal activity of DG70 and other anti-TB drugs. (A) Mid-log-phase cultures of BCG were incubated with the indicated compounds and compound combinations at 10× MIC of each compound. Aliquots of each culture were plated for CFU determination at the indicated time point and back calculated to CFU per milliliter. Dashed lines correspond to combination treatments with two drugs. (B) Highlight of panel A showing the time-kill kinetics of DG70 and PA824 alone and in combination. (C) Highlight of panel A showing the time-kill kinetics of DG70 and bedaquiline (BDQ) alone and in combination. (D) Highlight of panel A showing the time-kill kinetics of DG70 and isoniazid (INH) alone and in combination. Dimethyl sulfoxide (DMSO) was used as a vehicle to dissolve the compound. Means and standard deviations from three independent experiments are shown.

We studied whether DG70 was bactericidal and whether bactericidality could be increased by combining DG70 with other TB drugs. Log-phase *M. tuberculosis* cultures were treated with DG70 and other antitubercular agents, both individually and in combination at 10× their MIC. DG70 was highly bactericidal against BCG *in vitro*, leading to 5-log_10_ killing over 21 days. This activity was comparable to that of BDQ alone and better than that of INH or PA824 alone, especially as both drugs showed regrowth after 7 and 10 days, respectively (Fig. 8A). The bactericidal activity of DG70 could be reversed by pretreating the mycobacterial cultures with supplemental MK4. Interestingly, combining DG70 with other respiratory drugs such as BDQ or PA824 resulted in more rapid killing, producing sterile cultures (no CFU detected on plating) after 21 and 10 days of treatment, respectively (Fig. 8C and D). However, the most rapid killing was seen in the INH-DG70 combination, where mycobacterial cultures were reduced by 5 log_10_ CFU after only 2 days of treatment, compared to either INH or DG70 alone, where the same 2-day treatment reduced the cultures by only 1 log_10_ or less (Fig. 8B). Remarkably, the combination of INH and DG70 sterilized cultures after only 10 days of incubation.

### Validation and initial profiling for DG70.

DG70 was independently synthesized, and this new preparation exhibited whole-cell efficacy and Vero cell cytotoxicity that was similar to the GlaxoSmithKline (GSK)-sourced material. The synthesis of DG70 and analogs (Table 4) centered around construction of the carboxylic acid and amine coupling partners, ultimately joined under Schotten-Baumann conditions (Text S2). The compound exhibited modest kinetic solubility in pH 7.4 aqueous phosphate-buffered saline (PBS) (S = 0.66 µg/ml) but poor stability in the presence of a mouse liver microsomal preparation (half-life [*t*_1/2_] = 0.753 min, CL_int_ = 921 μl/min/mg protein). The major metabolite in this decomposition was determined to result from demethylation of both methyl ethers (Fig. S5). The MS data are consistent with the structure of bis(phenol) JSF-2950, which had significantly reduced whole-cell activity versus *M. tuberculosis* (MIC = 39 µg/ml). A focused set of DG70 analogs was prepared to further probe the antitubercular structure-activity relationships (SARs). Whole-cell efficacy depended on the presence of both methoxy groups in DG70 (cf. JSF-2907, -2971, and -2910), as well as the terminal methoxyphenyl moiety (cf. JSF-2926). The benzoyl moiety SAR was less straightforward. Removal of either the fluoro group (cf. JSF-2908) or both fluoro and chloro groups (cf. JSF-2912) led to small losses in activity, while excision of solely the chloro group (cf. JSF-2909) forfeited all efficacy. The amide carbonyl and amide N-H were also critical to whole-cell efficacy (cf. JSF-2951 and JSF-2949). To confirm MenG specificity, a subset of analogs was tested for MIC against DG70-resistant mutants, and these mutants were resistant to active DG70 analogs (Table S2).

10.1128/mBio.02022-16.2 TEXT S2  Schemes for synthesis of DG70 analogs. Synthesis of compounds via Schotten-Baumann reaction. Synthesis of compound JSF-2951. Download TEXT S2, DOCX file, 0.04 MB. Copyright © 2017 Sukheja et al. 2017 Sukheja et al. This content is distributed under the terms of the Creative Commons Attribution 4.0 International license.

10.1128/mBio.02022-16.7 FIG S5  Mouse liver microsome stability of DG70. Liquid chromatography-MS analysis demonstrated that the major mouse liver microsome-derived metabolite of DG70 involves didemethylation. Download FIG S5, DOCX file, 0.3 MB. Copyright © 2017 Sukheja et al. 2017 Sukheja et al. This content is distributed under the terms of the Creative Commons Attribution 4.0 International license.

10.1128/mBio.02022-16.9 TABLE S2  MICs of DG70 and its analogs in DG70-resistant mutants of H37Rv. Download TABLE S2, DOCX file, 0.01 MB. Copyright © 2017 Sukheja et al. 2017 Sukheja et al. This content is distributed under the terms of the Creative Commons Attribution 4.0 International license.

**TABLE 4:** Validation and initial probing of DG70 structure-activity relationships

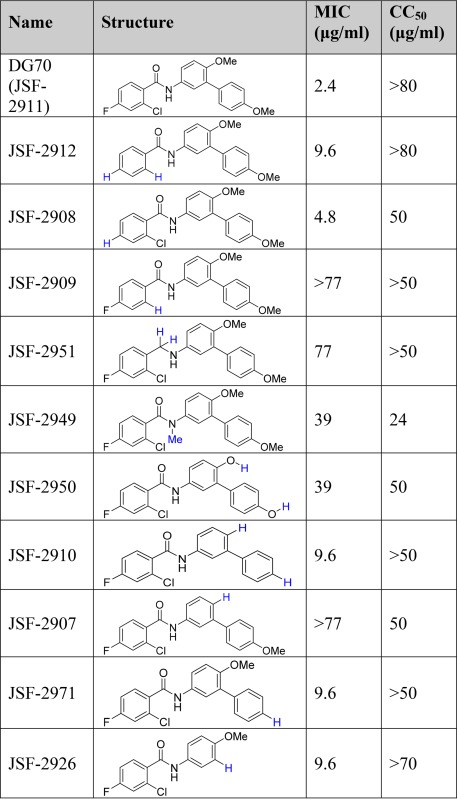

## DISCUSSION

We developed a novel whole-cell-based screen to detect inhibitors of the respiratory pathway, using induction of PcydAB as a highly specific reporter strain. This assay allowed us to identify DG70 as a potent inhibitor of both replicating and nonreplicating mycobacteria as well as drug-resistant *M. tuberculosis*. Our studies strongly suggest that the primary mechanism of action of DG70 is through inhibition of *M. tuberculosis* MenG, an essential enzyme involved in a critical terminal step of menaquinone biosynthesis. We isolated DG70-resistant mutants with SNPs in *menG*. Homology modeling and protein threading placed most of the mutations associated with resistance in close proximity either to the MenG binding site for *S*-adenosylmethionine, a cofactor used for methylation of DMK9 by MenG, or to the substrate site. Moreover, overexpression of mutant *menG* conferred high-level resistance on otherwise DG70-susceptible BCG strains. Finally, a combination of metabolic labeling and HRMS studies conclusively showed that DG70 inhibited the metabolic step catalyzed by MenG. DG70 treatment inhibited oxygen utilization in sealed mycobacterial culture tubes and significantly reduced ATP levels inside treated mycobacterial cells, further supporting the role of DG70 in inhibiting the respiratory chain.

Although earlier work has validated menaquinone biosynthesis as a druggable target in *M. tuberculosis*, DG70 is the first chemotype that specifically inhibits MenG (16, 29, 33), and our study is the first to show that this inhibition is highly bactericidal in *M. tuberculosis* persisters. DG70 showed several logs more activity against persisters than the currently licensed anti-TB drugs INH, RIF, and BDQ, and activity was also 80-fold higher than that of PA824. The increased effectiveness of DG70 in nonreplicating mycobacteria might be due to a lack of energy-generating pathways other than the respiratory chain in the nongrowing persistent mycobacteria (34). Menaquinone is known to be downregulated in persisters (16, 28), which could also result in heightened susceptibility to MenG inhibition. Although our data are promising and suggest that DG70 effectively kills nongrowing *M. tuberculosis*, it is important to note that the model that we used to measure killing in stationary phase requires culturability when the cultures are reexposed to conditions favorable for log-phase growth. Therefore, we cannot definitively determine whether the treated *M. tuberculosis* bacteria were killed as persisters under nongrowing conditions or whether they subsequently died when placed under growth-promoting conditions.

This work is also the first to note the highly desirable synergies between a menaquinone biosynthesis inhibitor and several important anti-TB drugs. DG70 and BDQ displayed almost identical killing curves *in vitro*. Both compounds showed delayed onset of bactericidal activity with less than 1-log_10_ killing in 4 days. However, they proved to be highly bactericidal, leading to a 5-log_10_ drop in CFU over the course of 21 days. Our results are consistent with previous findings that compounds which target the mycobacterial respiratory chain exhibit a delayed onset of bactericidal activity (23). Interestingly, combinations of DG70 and BDQ showed significantly better killing than did either compound by itself and completely sterilized cultures in 21 days. Furthermore, DG70 combined with either INH or PA824 was able to sterilize cultures in 10 days. This is particularly remarkable because it suggests that treatment with two drugs that target different enzymes can overcome both the persistence and the drug resistance that develop in the presence of single drug.

The activity and physiochemical and metabolic stability profiling of DG70 and its analogs support this biphenyl benzamide as a validated antitubercular hit. However, this series must be modified for improved metabolic stability, beginning by improving its stability in mouse liver microsomes. The identification of an inactive bis(phenol) metabolite and DG70 SAR and computational docking studies suggest an optimization route toward candidates for *in vivo* efficacy assessment. If developed into a drug, DG70 or another inhibitor of MenG with similar biological effects could become a highly effective component of TB therapy, with activity against both replicating and nonreplicating mycobacteria and considerable synergy with other anti-TB drugs.

## MATERIALS AND METHODS

### Bacterial strains.

*M. tuberculosis* H37Rv (ATCC 27294) and BCG (ATCC 35734) were used as reference strains. An avirulent Δ*panCD* Δ*leuCD* derivative of H37Rv (mc^2^6206) was a kind gift of William R. Jacobs, Jr., at the Albert Einstein College of Medicine, NY. The clinical *M. tuberculosis* strains tested were selected from collections of clinical isolates established by UNICEF, UNDP, the World Bank, and WHO Special Programs for Research and Training in Tropical Diseases (TDR). NTM and ESKAPE species and strains were *Mycobacterium fortuitum*, *Mycobacterium abscessus* (ATCC 19977), *Mycobacterium smegmatis* (ATCC 607), *Mycobacterium marinum* (ATCC 927), *Mycobacterium avium* subsp. *avium*, *Acinetobacter baumannii*, *Enterobacter cloacae*, *Enterococcus faecium*, *Klebsiella pneumoniae*, *Pseudomonas aeruginosa*, methicillin-resistant *Staphylococcus aureus* (MRSA), *Staphylococcus epidermidis* 14490, *Bacillus cereus*, *Burkholderia cepacia*, *Yersinia pseudotuberculosis*, *Brucella neotomae*, *Francisella philomiragia*, and *Legionella pneumophila*. All mycobacterial strains were maintained in Middlebrook 7H9 medium (Difco) supplemented with 0.5% (vol/vol) glycerol, 0.05% (vol/vol) Tween 80, and 10% (vol/vol) Middlebrook oleic acid-albumin-dextrose-catalase (OADC; Difco). Mycobacterial strains were plated on Middlebrook 7H10 (Difco) agar plates. When appropriate, hygromycin B (Roche) was added to the medium.

### Chemical and compound library.

A library of 168 structurally diverse small molecules with known whole-cell anti-*M. tuberculosis* activity was obtained from GlaxoSmithKline (GSK) (17). Antibiotics and MK4 were purchased from Sigma-Aldrich. Stock solutions were prepared in dimethyl sulfoxide (DMSO), and working stocks were diluted in the medium to 100 µM final concentrations. The MK9 standard was purchased from Santa Cruz Biotechnology.

### Construction of the CAW306 screening system.

The *mWasabi* gene was cloned into pMV306, a promoter-absent integrative vector, between HindIII and EcoRI restriction sites to generate pMV306*mWasabi*. The 489-bp sequence upstream of the translation start codon of the *cydA* gene was amplified from *M. tuberculosis* H37Rv genomic DNA using primers containing KpnI and HindIII restriction enzyme sites in the forward and reverse primers, respectively. The primer sequences were as follows: FP, 5′ GCCGGTACCTGTTGTTCATCAG 3′, and RP, 5′ GACAAGCTTCATCTGTCATCTCC 3′. The amplified product was then cloned between the KpnI and HindIII sites of pMV306*mWasabi* to generate the pMV306Pcyd*mWasabi* construct. After confirmation by DNA sequencing, the construct was electroporated into BCG and selected on 7H10 plates containing hygromycin B (50 µg/ml) to create the recombinant BCG strain CAW306. For screening, CAW306 was cultured to an optical density at 595 nm (OD_595_) of 0.2 in 7H9 medium supplemented with OADC and 0.02% Tween 80. This culture was then dispensed into 96-well black clear-bottom half-area plates (Corning) at 90 µl/well. Compounds from the test library were then added to plate wells in 10-µl aliquots for a final 10 µM test concentration. Positive and negative controls were included in each plate in triplicate. Plates were incubated for 24 h at 37°C, and the fluorescence of each well was recorded at an excitation (Ex) wavelength of 488 nm and an emission (Em) wavelength of 509 nm in a SpectraMax M5 microplate reader. Compounds were scored based on fold induction over vehicle. The library was screened in singlets due to limitations in the amount of compound available; however, each positive hit was tested in triplicate and then resupplied or synthesized. Z′ factor values were calculated for each plate using the mean and standard deviation of both positive and negative controls. The results from plates with a Z′ factor of ≥0.7 were deemed technically acceptable.

### MIC determination.

MICs of *M. tuberculosis*, BCG, NTM, and other bacterial isolates were measured in 96-well microtiter plates using a microdilution alamarBlue assay (MABA) as described previously (18). Briefly, bacterial cells grown to mid-log phase were diluted (1:100) in medium and 50 µl of this dilution was added to each well in 11 2-fold serial dilutions of test compound in the medium and then incubated for 7 days at 37°C. alamarBlue (Invitrogen) reagent was added along with 12.5 µl of 20% Tween 80 (Sigma) to evaluate bacterial cell viability. Plates were scanned after 24 h at 570-nm absorbance with a reference wavelength of 600 nm.

### Isolation of resistant mutants and their genetic characterization.

Mid-log-phase *M. tuberculosis* cultures (10^8^ CFU/ml) were plated on 7H10 agar containing DG70 at 4× and 8× MIC and incubated for 3 to 4 weeks at 37°C. Isolated colonies were purified and grown in liquid culture and subjected to MIC determination. Genomic DNA was isolated from confirmed resistant mutants and subjected to whole-genome sequencing using Illumina NextSeq at >100× coverage. Variant calling was performed by CLC Genomic Workbench 7.5 using the H37Rv sequence NC_000962.3 and the sequenced laboratory parent H37Rv strain as references (18).

### Construction of overexpression strains.

The *menG* gene was PCR amplified from the wild type as well as the DG70-resistant mutant of *M. tuberculosis* using primers FP (5′ aggcatatgagtcgcgccgcc 3′) and RP (5′ tccaagctttcactgcggggtttgtttgc 3′) and cloned into pMV306H-hsp60 (18) between NdeI and HindIII. The sequence-verified plasmids were transformed in BCG to create strains BCG::pMV306PHsp60, BCG::pMV306PHsp60-menG_Wt, BCG::pMV306PHsp60-menG_F118L, and BCG::pMV306PHsp60-menG_V20A.

### Cytotoxicity testing.

Vero cells (ATCC CCL-81) were used to test cytotoxicity as described previously (18). Briefly, Vero cells were plated at a concentration of 10^5^ cells/well in 96-well plates and incubated for 2 to 3 h to allow the cells to settle. Test compounds were serially diluted separately in Eagle’s minimal essential medium to generate test concentrations ranging from 200 to 0.1× *M. tuberculosis* MIC. The serial dilutions were then added to the plated cells and incubated for 48 h at 37°C. The viability of Vero cells exposed to each compound was determined using an MTT [3-(4,5-dimethyl-2-thiazolyl)-2,5-diphenyl-2H-tetrazolium bromide] cell viability kit (Promega).

### Determination of compound interactions by checkerboard assay.

We used a checkerboard assay to test for interactions between DG70 and drugs with known anti-TB activity using alamarBlue as a viability marker (35). Drug combinations were categorized based on a FIC of ≤0.5 as synergism, a FIC of >0.5 and ≤1 as additivity, a FIC of >1 and <4 as no interaction, and a FIC of ≥4 as antagonism (36).

### Determining bactericidal concentration in a nutritionally deprived persistence model.

Exponentially growing cultures of *M. tuberculosis* at an OD_600_ of 0.4 to 0.6 were harvested by centrifugation (3,000 × *g*, 10 min), washed twice with PBS (Invitrogen) supplemented with 0.025% Tween 80, and incubated in this nutritionally deprived medium for 6 weeks at 37°C. The bactericidal activity of each compound and compound combination against the resulting nongrowing bacilli was determined by exposing each culture to compounds for 7 days at 37°C and then measuring CFU by plating appropriate dilutions in 7H9 medium on 7H11 agar plates (37).

### Twenty-one-day kill kinetics.

Mid-log-phase BCG cultures were diluted in fresh medium to an OD_600_ of 0.1 to 0.2. Compounds were then added to culture aliquots at 10× MIC of each compound. Dilutions of each culture were plated at selected intervals on 7H10 agar plates to determine CFU.

### Intracellular activity in J774A.1 murine macrophages.

Intracellular activity was evaluated in J774A.1 (ATCC 113-67) cells as described previously (18). Briefly, J774A.1 cells were maintained in Dulbecco’s minimal essential medium (DMEM) supplemented with 10% heat-inactivated fetal bovine serum (FBS). The cells were placed into 96-well tissue culture white clear-bottom plates (Corning) in a 100-µl volume containing 2 × 10^4^ cells per well and allowed to adhere overnight. The avirulent Δ*panCD* Δ*leuCD M. tuberculosis* strain mc^2^6206 was cultured to mid-log phase; diluted in DMEM supplemented with FBS, pantothenic acid, and leucine; and added to the adherent macrophages at 10^4^ cells/well. The assay plates were then incubated for 4 h to allow bacteria to infect the macrophages. The remaining extracellular bacteria were then removed by replacing the medium with 100 µl of DMEM containing 50 µg/ml of gentamicin and incubating it for 1 h. The infected cells were then washed twice with 100 µl of DMEM supplemented with FBS, pantothenic acid, and leucine, and the final wash was replaced with 100 µl of complete DMEM containing the indicated concentrations of each test compound. Each compound concentration was tested in triplicates. On day 3, cells were washed with DMEM, lysed with distilled water (dH_2_O), serially diluted, and plated for CFU.

### ATP determination.

BCG was grown to an OD_595_ of 0.3. DG70 and other control drugs were added at selected concentrations. The cultures were then incubated at 37°C for 3 days. Intracellular ATP was quantified using a BacTiter Glo microbial cell viability assay kit (Promega) as previously described (23). ATP standards ranging from 0.1 to 10,000 nM were also included.

### Oxygen consumption.

The effect of DG70 on *M. tuberculosis* oxygen consumption was tested using a methylene blue decolorization assay as previously described (19). Decolorization was monitored by measuring absorbance at 6, 12, and 24 h at 665 nm. In the rescue experiment, the indicated concentration of MK4 was added prior to the inhibitor.

### Menaquinone analysis by whole-cell labeling.

Five-milliliter cultures of BCG were grown to an OD_595_ of 0.6, pelleted, and resuspended in 1/10 fresh medium, and 0.5-ml subsets were incubated with compounds for 1 h prior to the addition of 5 µCi l-[*methyl*-^14^C]methionine (American Radiolabeled Chemical) and further incubated for 4 h. Neutral lipids were then extracted (16), dissolved in hexane, applied to silica gel TLC plates, and developed using hexane-diethyl ether (95:5, vol/vol).

### Identification of menaquinone.

Ten-milliliter cultures of BCG were grown in 7H9 medium containing 0.05% Tween to mid-log phase and treated with compounds for 5 generations (3 days). Neutral lipids were then extracted as described above and analyzed by high-resolution mass spectrometry on a Bruker Daltonics mass spectrometer with atmospheric pressure chemical ionization as the ionization interface (16).
