# Supplementary material for: A Novel Small-Molecule Inhibitor of the Mycobacterium tuberculosis Demethylmenaquinone Methyltransferase MenG Is Bactericidal to Both Growing and Nutritionally Deprived Persister Cells
Source: mBio. 2017 Feb 14;8(1):e02022-16. doi: 10.1128/mBio.02022-16 (PMC5312080; doi:10.1128/mBio.02022-16)
Supplement: FIG S1 [file mbo001173186sf1.docx]

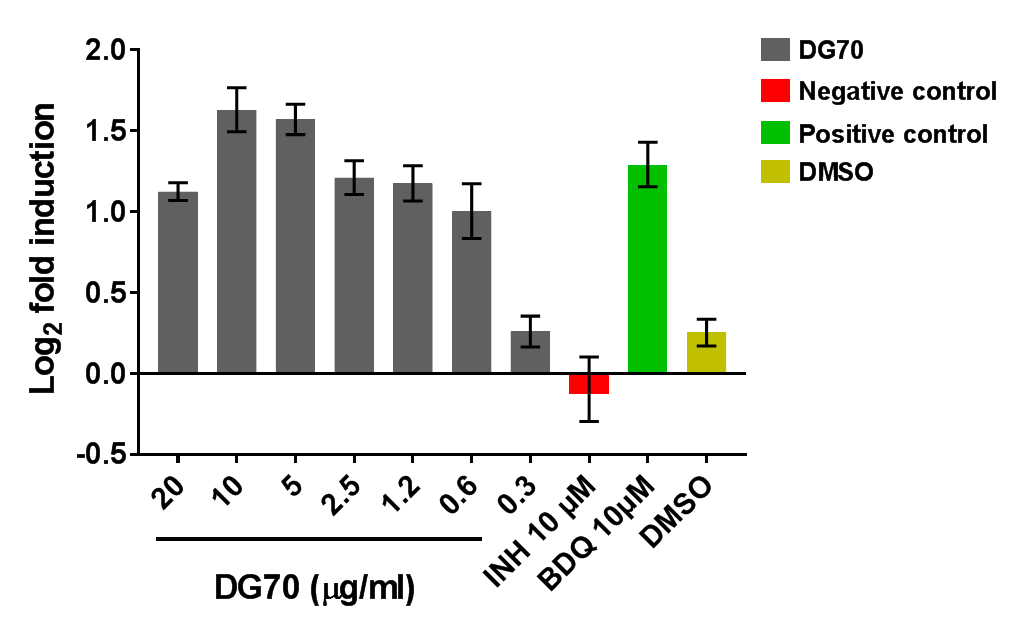


**Figure S1: PcydAB induction dose response for DG70**. PcydAB fold induction over vehicle dose response profile of BCG reporter strain treated with 2 fold serial dilution of DG70 fluorescence assay at maximum of 8x the MIC in 96 well plate format. isoniazid (INH) was used as negative control, bedaquiline (BDQ) was used as positive control and DMSO was the vehicle used to dissolve compound.
