# Supplementary material for: A Novel Small-Molecule Inhibitor of the Mycobacterium tuberculosis Demethylmenaquinone Methyltransferase MenG Is Bactericidal to Both Growing and Nutritionally Deprived Persister Cells
Source: mBio. 2017 Feb 14;8(1):e02022-16. doi: 10.1128/mBio.02022-16 (PMC5312080; doi:10.1128/mBio.02022-16)
Supplement: TEXT S1 [file mbo001173186s1.docx]

**A Novel Small-Molecule Inhibitor of the *Mycobacterium tuberculosis* Demethylmenaquinone Methyltransferase MenG Is Bactericidal to Both Growing and Nutritionally Deprived Persister Cells**

Paridhi Sukheja,^a^ Pradeep Kumar,^a^ Nisha Mittal,^b^ Shao-Gang Li,^b^ Eric Singleton,^a^ Riccardo Russo,^a^ Alexander L. Perryman,^b^ Riju Shrestha,^a^ Divya Awasthi,^b^ Seema Husain,^d,e^ Patricia Soteropoulos,^d,e^ Roman Brukh,^c^ Nancy Connell,^a^  Joel S. Freundlich,^a,b^ David Alland^a*^

Division of Infectious Disease, Department of Medicine, and the Ruy V. Lourenço Center for the Study of Emerging and Reemerging Pathogens, Rutgers University-New Jersey Medical School, Newark, NJ, USA^a^; Department of Pharmacology, Physiology, and Neuroscience, Rutgers University-New Jersey Medical School, Newark, NJ, USA^b^; Department of Chemistry, Rutgers University-Newark College of Arts and Sciences, Newark, NJ, USA^c^; Genomics Center, Rutgers University-New Jersey Medical School, Newark, NJ, USA^d^; Department of Microbiology, Biochemistry and Molecular Genetics, Rutgers University-New Jersey Medical School, Newark, NJ, USA^e^

*Corresponding author : David Alland (david.alland@rutgers.edu)

**Supplementary Material**

**COMPOUND SYNTHESIS:**

**General Procedure A** (Synthesis of compounds DG70, JSF-2907 – JSF-2910, JSF-2912, JSF-2926 & JSF-2971): To a solution of compound 4'-methoxybiophenyl-3-yl) amine hydrogen chloride salt; **A1** (0.5 mmol, 0.117 g) in dichloromethane (0.1 M, 5.0 mL), 1 M NaOH_(aq)_ (0.5 mL) was added. To the reaction mixture 2-chloro-4-flurobenzoyl chloride; **A2** (0.5 mmol, 0.067 mL) was added and the resulting mixture was stirred for one hour. After completion, the reaction mixture was extracted with ethyl acetate (2 x 30 mL), the combined organic layers were washed with saturated aqueous brine solution (1 x 20 mL), dried over anhydrous sodium sulfate and concentrated under reduced pressure. The crude mixture was purified by flash chromatography on silica gel using 5% EtOAc in dichloromethane as an eluent to obtain the desired compound in 84% yield.

**Synthesis and Characterization Data of Compounds:**

**2-chloro-N-(4',6-dimethoxy-[1,1'-biphenyl]-3-yl)-4-fluorobenzamide (DG70**)**:**

Synthetic **DG70** was prepared following the general procedure **A** and was obtained as a light pink solid in 84% (0.161 g) yield: ^1^H NMR (500 MHz, d_6_-DMSO) δ 10.4 (br s, 1), 7.70 – 7.64 (m, 1), 7.62 (br s, 1), 7.61 – 7.59 (m, 1), 7.59 – 7.54 (m, 1), 7.39 (d, *J* = 8.3 Hz, 2), 7.34 (app td, *J* = 8.5, 1.3 Hz, 1), 7.07 (d, *J* = 8.9 Hz, 1), 6.98 (d, *J* = 8.3 Hz, 2), 3.78 (s, 3), 3.74 (s, 3); m/z 387.1 [M + 2H]^+^.

**N-([1,1'-biphenyl]-3-yl)-2-chloro-4-fluorobenzamide (JSF-2910**)**:** **JSF-2910** was prepared following the general procedure **A** using the corresponding amine and was obtained as a white solid in 80% (0.129 g) yield: ^1^H NMR (500 MHz, d_6_-DMSO) δ 10.6 (br s, 1), 8.04 (s, 1), 7.67 - 7.74 (comp, 2), 7.61 - 7.65 (comp, 2), 7.60 (br s, 1), 7.49 (t, *J* = 7.8 Hz, 2), 7.45 (d, *J* = 7.63 Hz, 1), 7.33 - 7.43 (comp, 3); m/z 326.1 [M + H]^+^.

**N-(4',6-dimethoxy-[1,1'-biphenyl]-3-yl)benzamide (JSF-2912**)**:** **JSF-2912** was prepared following the general procedure **A** using the corresponding amine and was obtained as a pinkish white solid in 80% (0.129 g) yield: ^1^H NMR (500 MHz, d_6_-DMSO) δ 10.2 (br s, 1), 7.96 (d, *J* = 8.1 Hz, 2), 7.76 – 7.67 (comp, 2), 7.58 (ap t, *J* = 7.0 Hz, 1), 7.52 (ap t, *J* = 7.5 Hz, 2), 7.43 (d, *J* = 8.3 Hz, 2), 7.08 (d, *J* = 8.7 Hz, 1), 6.98 (d, *J* = 8.4 Hz, 2), 3.79 (s, 3), 3.75 (s, 3); m/z 334.2 [M + H]^+^.

**2-chloro-4-fluoro-N-(6-methoxy-[1,1'-biphenyl]-3-yl)benzamide (JSF-2971**)**:** **JSF-2971** was prepared following the general procedure **A** using the corresponding amine and was obtained as a white solid in 70% (0.124 g) yield: ^1^H NMR (500 MHz, d_6_-DMSO) δ 10.4 (br s, 1), 7.70 - 7.63 (comp, 3), 7.61 - 7.55 (m, 1), 7.46 (d, *J* = 7.8 Hz, 2), 7.42 (t, *J* = 7.4 Hz, 2), 7.37 - 7.31 (comp, 2), 7.11 (d, *J* = 9.4 Hz, 1), 3.75 (s, 3); m/z 356.1 [M + H]^+^.

**2-chloro-4-fluoro-N-(4-methoxyphenyl)benzamide (JSF-2926):** **JSF-2926** was prepared following the general procedure **A** using the corresponding amine and was obtained as a white solid in 96% (0.074 g) yield: ^1^H NMR (500 MHz, d_6_-DMSO) δ 10.3 (s, 1), 7.65 (dd, *J* = 8.6, 6.2 Hz, 1), 7.63 – 7.59 (m, 2), 7.57 (dd, *J* = 9.0, 2.5 Hz, 1), 7.34 (td, *J* = 8.5, 2.5 Hz, 1), 6.95 – 6.90 (m, 2), 3.74 (s, 3); m/z 280.0 [M + H]^+^.

**2-chloro-4-fluoro-N-(4'-methoxy-[1,1'-biphenyl]-3-yl)benzamide** **(JSF-2907):** **JSF-2907** was prepared following the general procedure **A** using the corresponding amine and was obtained as a white solid in 53% (0.094 g) yield: ^1^H NMR (500 MHz, d_6_-DMSO) δ 10.6 (s, 1), 7.98 (s, 1), 7.70 (t, *J* = 7.2 Hz, 1), 7.64 (d, *J* = 7.7 Hz, 1), 7.60 (d, *J* = 9.0 Hz, 1), 7.56 (d, *J* = 8.0 Hz, 2), 7.41 (t, *J* = 7.6 Hz, 1), 7.37 (t, *J* = 7.4 Hz, 2), 7.05 (d, *J* = 8.0 Hz, 2), 3.80 (s, 3); m/z 356.0 [M + H]^+^.

**2-chloro-N-(4',6-dimethoxy-[1,1'-biphenyl]-3-yl)benzamide (JSF-2908**)**:** **JSF-2908** was prepared following the general procedure **A** using the corresponding acid chloride and was obtained as an off white solid in >99% (0.070 g) yield: ^1^H NMR (500 MHz, d_6_-DMSO) δ 10.4 (s, 1), 7.66 – 7.61 (m, 2), 7.57 (t, *J* = 7.9 Hz, 2), 7.50 (t, *J* = 7.7 Hz, 1), 7.45 (t, *J* = 7.3 Hz, 1), 7.40 (d, *J* = 7.7 Hz, 2), 7.08 (d, *J* = 8.6 Hz, 1), 6.99 (d, *J* = 7.7 Hz, 2), 3.79 (s, 3), 3.74 (s, 3); m/z 368.0 [M + H]^+^.

**N-(4',6-dimethoxy-[1,1'-biphenyl]-3-yl)-4-fluorobenzamide (JSF-2909**)**:** **JSF-2909** was prepared following the general procedure **A** using the corresponding acid chloride and was obtained as an off white solid in 83% (0.055 g) yield; ^1^H NMR (500 MHz, d_6_-DMSO) δ 10.2 (s, 1), 8.11 – 7.94 (m, 2), 7.70 (d, *J* = 8.9 Hz, 1), 7.68 (s, 1), 7.42 (d, *J* = 7.7 Hz, 2), 7.36 (t, *J* = 8.3 Hz, 2), 7.08 (d, *J* = 8.7 Hz, 1), 6.99 (d, *J* = 7.7 Hz, 2), 3.79 (s, 3), 3.75 (s, 3); m/z 352.0 [M + H]^+^.

**2-chloro-N-(4',6-dimethoxy-[1,1'-biphenyl]-3-yl)-4-fluoro-N-methylbenzamide (JSF-2949**): To a suspension of NaH (2.0 equiv, 0.14 mmol, 3.3 mg) in THF (1 mL) was added a solution of JSF-2911 in 1.0 mL THF at 0 °C. The resulting solution was stirred for 15 minutes at 0 °C, followed by addition of methyl iodide (2.0 equiv, 0.14 mmol, 8.6 μL). The reaction mixture was stirred at room temperature for 2 hours. After completion, the reaction mixture was quenched with sat. NH_4_Cl and extracted with EtOAc (2 x 20 mL), the combined organic layers were washed with saturated aqueous brine solution (1 x 20 mL), dried with sodium sulfate and concentrated under reduced pressure. The crude mixture was purified by flash chromatography on silica gel using 5% EtOAc in dichloromethane as an eluent to obtain **JSF-2949** as a colorless oil in 70% yield (0.028 g): ^1^H NMR (500 MHz, d_6_-DMSO) δ 7.29 (d, *J* = 8.8 Hz, 2), 7.23 – 7.16 (m, 1), 7.07 (br s, 1), 7.06 – 6.98 (comp, 2), 6.95 (d, *J* = 8.1, 2), 6.86 (app t, *J* = 8.2 Hz, 1), 6.77 (d, *J* = 8.7 Hz, 1), 3.86 (s, 3), 3.76 (s, 3), 3.51 (s, 3); m/z 401.1 [M + 2H]^+^.

**2-chloro-N-(4',6-dihydroxy-[1,1'-biphenyl]-3-yl)-4-fluorobenzamide (JSF-2950**): **JSF-2950** was prepared following a reported procedure (using JSF-2911 as the starting material. The desired compound was obtained as a light brown solid in 75% yield (0.048 g): ^1^H NMR (500 MHz, d_6_-DMSO) δ 10.3 (br s, 1), 9.40 (br s, 1), 9.3 (br s, 1), 7.68 – 7.61 (m, 1), 7.60 – 7.52 (comp, 2), 7.41 (d, *J* = 8.7 Hz, 1), 7.37 – 7.28 (comp, 3), 6.86 (d, *J* = 8.4,1), 6.79 (d, *J* = 7.9 Hz, 2); m/z 359.1 [M + 2H]^+^.

**N-(2-chloro-4-fluorobenzyl)-4',6-dimethoxy-[1,1'-biphenyl]-3-amine (JSF-2951**): To a suspension of A1 (0.3 mmol, 0.080 g) and A3 (0.3 mmol, 0.047 g) in methanol (3.0 mL) was added NaBH_4_ (5.0 equiv, 1.5 mmol, 0.056 g) at room temperature **(Scheme S2).** The resulting solution was stirred for 8 hours. After completion, the reaction mixture was quenched with sat. NH_4_Cl_(aq)_ and extracted with EtOAc (2 x 20 mL). The combined organic layers were washed with saturated aqueous brine solution (1 x 20 mL), dried over anhydrous sodium sulfate and concentrated under reduced pressure. The crude mixture was purified by flash chromatography on silica gel using 5% EtOAc in dichloromethane as an eluent to obtain **JSF-2951** as a yellow oil in 65% yield (0.072 g): ^1^H NMR (500 MHz, d_6_-DMSO) δ 7.53 – 7.41 (comp, 3), 7.28 (br s, 1), 7.15 (d, *J* = 8.5, 1), 7.01 – 6.91 (comp, 3), 6.86 (d, *J* = 8.9 Hz, 1), 6.72 – 6.66 (m, 1), 6.60 (d, *J* = 9.0 Hz, 1), 4.41 (s, 2), 3.86 (s, 3), 3.73 (s, 3).; m/z 373.1 [M + 2H]^+^.

**COMPUTATIONAL METHODS:**

**Sequence Analyses**

The FASTA-formatted sequence of MenG (from *M. tuberculosis* H37Rv Rv0558; also known as MenH or UbiE) was obtained from the TubercuList server (1). This sequence was analyzed with JPred4 (2) TMHMM transmembrane prediction server using Hidden Markov Models,(3) Pfam search using Hidden Markov Models (hmmsearch) on the EMBL server (https://www.ebi.ac.uk),(4-6) and PsiPRED4.(7, 8) Additional sequence searches for homologs with known crystal structures were performed on the Protein Data Bank (PDB; http://www.rcsb.org), using the advanced search, by sequence tool, with either BLAST or PSI-BLAST and the default run parameters (9-12).

**Homology Modelling and Protein Threading**

Despite a large number of related homologs in the protein sequence databases, no closely related homolog had a structure available. Of the structures available in the PDB, most proteins had <30% sequence identity and only had homology with less than half of the MenG sequence. The few proteins that had close to or >30% similarity for a majority of the MenG sequence belonged to a methyltransferase class of enzymes, and they were selected as templates. The templates selected were as follows (listed according to their PDB ID, with % identity and similarity values from Discovery Studio 4.5, using the BLOSUM 30 pairwise scoring matrix, followed by % identity and % similarity values from the SWISS MODEL results in parentheses): 4obw, which displays 27.2% sequence identity and 43.3% sequence similarity overall (35.5% identity and 37% similarity for residues 20-227 of MenG); 3bus, which has 22.7% sequence identity and 36.9% sequence similarity overall (24.6% identity and 31% similarity for residues 27-216 of the query); 3ou2, which also has 22.7% sequence identity and 36.9% sequence similarity (21.5% identity and 29% similarity for residues 10-214 of MenG); and 2avn, which displays 21.9% sequence identity and 36.3% sequence similarity (28.3% identity and 31% similarity, for residues 15-144 of MenG).

Since none of the four residues that mutate upon treatment with DG70 are near the dimeric interface (according to inspection of the corresponding positions in homologs that have known crystal structures available), and since each monomer has a complete active site, we chose to model this system as a monomer (**Figure S3A and S3C**). Due to the very low sequence identity between MenG and the available templates, models of MenG were created using several different methods and algorithms. The SWISS MODEL server (13) was used to create four different homology models, based on three different templates (PDB ID: 3bus, 3ou2, and two from 4obw) (14-16). The pDomTHREADER and pGenTHREADER protocols on PsiPRED4 were used to create models based on CATH domain 1im8A00 (of a methyltransferase) and PDB ID: 4obx, respectively (16-19) The multiple sequence alignment protocol in Discovery Studio 4.5(BIOVIA, San Diego, CA) was used with four different templates (PDB ID: 4obw, 2avn, 3bus, and 3ou2 to create a set of 20 homology models with MODELLER, from which the two models with the best DOPE scores (discrete optimized protein energy) were selected. All MenG models and the templates were superimposed onto a single coordinate reference frame, using the align by alpha carbon command in PyMOL.(The PyMOL Molecular Graphics System, Version 1.8 Schrödinger, LLC) Hydrogen atoms were added to the MenG models and the templates using the MolProbity server, with the default parameters, including the electron cloud-based method and allowing the ability of His, Asn, and Gln side-chains to flip if doing so lowered the energy of the system All MenG models were minimized in Discovery Studio 4.5,(BIOVIA, San Diego, CA) using the “simple minimization” protocol, the CHARMm force-field, the Generalized Born implicit solvent model, and a spherical cutoff for electrostatics calculations.

**Docking**

The sdf file of DG70 (with all hydrogen atoms) was prepared in and downloaded from our vault on CDD (Collaborative Drug Discovery; <http://www.collaborativedrug.com>). Models of SAM, SAH, and SAI had their hydrogen atoms added by MolProbity when that server was used to protonate the crystal structures of the templates. Additional models of DG70 were prepared by minimizing them in either Discovery Studio 4.5(BIOVIA, San Diego, CA) using their implementation of the MMFF force-field or in Avogadro with the MMFF94s force-field(DOI**:** 10.1002/(SICI)1096-987X(199604)17:5/6<490::AID-JCC1>3.0.CO;2-P), using the steepest descent method, followed by the conjugate gradient method, until convergence occurred. The original model and the two different minimized outputs thus produced 3 different models of DG70. An additional 3 models were produced by allowing the amide bond in the ligand model to freely rotate during the docking studies (due to the potential issues associated with docking to rigid targets and/or to homology models based on templates with low sequence identity), to produce a final set of 6 different ligand models of DG70.

All of the template crystal structures, the original MenG models, and the minimized MenG models were used as targets for the docking studies (to create a set of 16 different targets). The pdbqt docking input format for the ligands, the homology models, and all of the templates were visually inspected with the Python Molecular Viewer (PMV 1.5.6) and prepared with AutoDockTools (ADT 4.2) Docking calculations were performed with AutoDock Vina 1.1.2. To enable using a single “grid box” (region in which the ligands initially explore during docking) for all of the MenG models and templates, a large grid box was utilized: 30 x 40 x 30 (x, y, z size in Angstroms). The grid box had a center near the middle of the crystallographic conformations of SAM, SAH, and SAI in the aforementioned templates. Since a large grid box was utilized, the “exhaustiveness” setting was increased to 20. Docking calculations were performed on a Linux workstation (Precision T7600 with 16GB of RAM and 12 Xeon CPU’s at 2.30 GHz) running Ubuntu. The top and second-ranked docked modes / ligand model were analyzed and visually inspected using PMV1.5.6, which was also utilized to create molecular images of the docking results (20). Key factors that were judged favorably during the visual inspection process were (a) consistency in the predicted binding modes when different, independent models of DG70 were docked to a target, (b) forming favorable interactions with the four residues that are known to mutate during the selective pressure applied by DG70 treatment, and (c) the general visual inspection rules that we have described in previous publications

**COMPUTATIONAL RESULTS:**

**Additional Sequence Analysis**

According to the JPred4 sequence alignment with the most similar homologs, three of the four residues that mutate under the selective pressure provided by DG70 treatment are highly conserved. Val20 was a Val in the corresponding position for 67 of the 100 most closely related sequences, it was the similar residue Ile in 32 / 100, and it was a Thr in only 1 of the 100 closest homologs. An Ala was not detected at that corresponding position until the 234^th^ most closely related homolog, which corresponds to UniRef90 i1d192, a methylase involved in ubiquinone/menaquinone biosynthesis from *Saccharomonospora glauca* K62, a type of Actinobacterium. Similarly, Phe118 was a Phe in the corresponding position for 95 of the 100 most closely related sequences, it was the similar residue Tyr in 4 / 100, and it was a Thr in 1 / 100. A Leu was not detected at this position until the 213^th^ most closely related sequence, which was UniRef90 e8t363, a demethylmenaquinone methyltransferase from *Thermovibrio ammonificans*. At the position corresponding to Ser188 in MenG, there was a Ser in 95 of the 100 most closely related sequences, with the similar residue Thr in the other 5 / 100. An Ala was not detected until the 250^th^ most closely related sequence, which was UniRef90 u1psh0, a methylase involved in ubiquinone/menaquinone biosynthesis from *Haloquadratum walsbyi* (a halobacterium from the *Archaea* lineage). Trp75 was the exception: among the 100 closest homologs, the position corresponding to Trp75 was a Trp in only 7 / 100 sequences. In the order of their initial appearance in the alignment, at this position His (also in 7 / 100), Ser (9 / 100), Arg (14 / 100), Glu (26 / 100), Lys (13 / 100), Met (1 / 100), Asn (5 / 100), Gln (11 / 100), Thr (3 / 100), Pro (1 / 100), Asp (2 / 100), and Ala (1 /100) were all found in the top 100 most closely related sequences, with Ala first being detected in the 95^th^ most closely related homolog.

**Positive control docking results**

To support the feasibility of using AutoDock Vina docking calculations against this system, positive control re-docking and cross-docking studies were first performed. When the co-substrate SAM (S-adenosyl-L-methionine) was re-docked against PDB ID: 4obw, the heavy atom RMSD (Root Mean Square Deviation) between the docked and crystallographic binding modes was 1.04 Å^2^. An RMSD ≤ 2.0 Å^2^ is generally considered accurate within the docking field. When the model of the co-product SAH (S-adenosyl-L-homocysteine, the de-methylated version of SAM) from PDB ID: 3ou2 was docked against PDB ID: 2avn, the docked and crystallographic binding modes displayed an RMSD of 0.64 Å^2^. When SAI (S-adenosyl-L-homoselenocysteine) was used in these positive controls, the selenium atom was converted to a sulfur (to make it SAH) to enable the docking calculations; it produced an RMSD of 0.97 Å^2^ when it was re-docked to the PDB ID: 2avn target of the template. Since these calculations produced RMSD values ≤ 1.0 Å^2^, the positive control docking studies were successful.

**Detailed docking results against the MenG models**

Since the available templates for constructing the MenG models were all in the “twilight region” of sequence identity, significant weight cannot be given to the accuracy of any particular MenG model that was produced. However, it is possible that the docking results against the entire collection of different MenG models and the templates could provide insight regarding the potential types of interactions that DG70 perhaps displays with ubiquinone/menaquinone biosynthesis methyltransferases*.* Slight differences in the side-chain conformations of a target can cause large differences in docking results. The different MenG models displayed large differences in both backbone and side-chain conformations, which is why an ensemble of targets was utilized and recurring trends were given extra weight. In most of the models, part of DG70 docked within the SAM binding site and/or the substrate site (i.e., according to the annotation on UniProt, which indicates that Asp100 and Ala101 are part of this cofactor site, while Thr62, Asp80, and Ser117 were listed as being part of the substrate site).

A convergent aspect of these results was that the types of pi-pi stacking interactions observed in the docking results against the templates from PDB ID: 3ou2 and 2avn were also frequently observed when the six slightly different models of DG70 were docked against the collection of different MenG models. Although several different types of predicted binding modes for DG70 seem plausible, the top docking results all formed strong interactions with multiple residues that mutate in *M. tuberculosis* MenG under the selective pressure provided by DG70 treatment. The aromatic rings of DG70 were often predicted to form base stacking or T-stacking interactions with Phe118 and/or Trp75. The methyl of a methoxy group was predicted to form favorable hydrophobic packing interactions with Val20 in many of the docking results. In other results the oxygen of a methoxy group formed favorable electrostatics interactions with Ser188. The amino group of the peptide linker often formed a hydrogen bond with the carboxylate group in the side-chain of Asp7 (which mutates in *BCG* when it is treated with DG70). Cation-pi interactions were also frequently observed between the 2-chloro-4-fluoro-phenyl ring and the side-chain of Arg121 when docking DG70 to several of the MenG targets.

In one mode (against the minimized version of the MenG homology model from MODELLER that had the 2^nd^ highest DOPE score), the terminal methoxy phenyl ring was predicted to form base stacking interactions with Phe118, the central methoxy phenyl ring packed favorably with the key residue Val20, and the 2-chloro-4-fluoro-phenyl ring displayed T-stacking interactions with Trp75. In a mode produced against the un-minimized, original MenG model from MODELLER with the 2^nd^ highest DOPE score, the terminal methoxy phenyl ring was also predicted to form base stacking interactions with Phe188, but in this mode the central methoxy phenyl ring formed T-stacking interactions with Trp75 (see **Figure S3. D**). The amino group of the peptide bond formed a hydrogen bond with the carboxylate group of Asp7 (which was observed in many docked modes against many target models), and the chlorine atom of the 2-chloro-4-fluoro-phenyl ring formed favorable electrostatics interactions with the side-chain of Arg121. Two other two-ranked docked modes against this target produced very similar predicted binding modes.

Another docked mode was produced by docking DG70 (the ligand model that was minimized with Avogadro prior to docking, and for which the peptide bond was held rigid in the conventional trans conformation) against the MenG homology model from MODELLER that had the highest DOPE score. Similar types of interactions were observed, but different parts of the ligand were sometimes involved in them (see **Figure S3. B**). The central methoxy phenyl ring was predicted to form favorable T-stacking interactions with Trp75. The amino group of the peptide bond formed a hydrogen bond with the side-chain of Asp7, and the carbonyl formed favorable electrostatics interactions with the side-chain of Arg121. The 2-chloro-4-fluoro-phenyl ring formed favorable pi-cation stacking interactions with the side-chain of Arg121, and the chlorine atom of this ring formed a halogen bond with the backbone carbonyl of Ala181. This binding mode was similar to a mode predicted in docking studies against the minimized version of this MenG homology model that had the highest DOPE score. But against the minimized version of the target, halogen bonds were formed with the backbone carbonyl of Phe118 and of Pro10, instead of with Ala181.

In one docked mode against the MenG model produced by threading its sequence onto the 1im8A00 CATH domain, the terminal methoxy phenyl ring of DG70 was predicted to form base stacking interactions with Phe118, and the methyl of that methoxy has favorable hydrophobic packing with Val20. The input ligand model that produced this result was minimized with Avogadro prior to the docking studies, and its peptide bond was held rigid in the conventional trans conformation during the docking calculations. The oxygen atom in the methoxy group attached to the central phenyl ring formed favorable electrostatic interactions with the hydroxyl in the side-chain of Ser188 (see **Figure S3.** **F)**. If this key residue Ser188 changed its rotamer (i.e., sampled small changes in the chi1 and chi2 torsion angles in its side-chain), then that interaction could perhaps become a hydrogen bond. This oxygen atom was also predicted to form a hydrogen bond with the side-chain of Arg121. Removing this central methoxy group from the scaffold (to generate compound JSF-2907) destroyed the whole-cell activity of the analog, which adds support to the predictions that this methoxy group forms strong interactions. The carbonyl of the peptide bond linker was predicted to form a hydrogen bond with the side-chain of Asn122 and also favorable electrostatics interactions with the side-chains of Arg121 and Arg11. The fluorine atom of the other terminal phenyl ring could perhaps form a halogen bond with the side-chain of Asp80. In additional docking studies against this target model, the top-ranked modes from two different ligand models (i.e., the output from energy minimization with Avogadro and the minimized output from Discovery Studio, for which the peptide bonds of both models were allowed to freely rotate during the docking calculations) produced identical binding modes and scores: the central methoxy phenyl ring of DG70 was predicted to base stack with Phe118, and the methyl of the methoxy group attached to that ring was predicted to form favorable hydrophobic packing with Val20 (see **Figure S3. E**). In this docked mode, the carbonyl of the amide bond also formed a hydrogen bond with the side-chain of Arg121. The methyl group of the terminal methoxy phenyl ring formed favorable hydrophobic packing with the side-chain of Leu164, and the oxygen atom of that methoxy group formed favorable electrostatics interactions with the side-chains of Lys23 and Arg173. The side-chain of Lys23 also formed favorable cation-pi interactions with that ring.

Unlike the aforementioned results, several targets did not produce meaningful docking results for DG70, based on knowledge of the active sites of these enzymes and, more importantly, on the residues known to mutate under the selective pressure provided by DG70 treatment. The four SWISS MODEL-based homology models of MenG that used the PDB ID: 4obw as a template (i.e., two outputs from SWISS MODEL, of which the original and the minimized versions were used as targets) produced docking results in which all six models of DG70 docked onto the surface of the target, instead of docking within the active site. The same trend occurred when the original PDB ID: 4obw crystal structure of the template or the original PDB ID: 3bus crystal structure of the template were used as targets for DG70. However, when the MenG sequence from *M. tuberculosis* was threaded onto the other crystal structure of a template (PDB ID: 4obx), one of the top-ranked modes passed visual inspection. But most of the docking results against this target produced unsatisfactory modes in which the ligand was docked to the surface of the protein. The best binding mode produced against this threading-based model is displayed in **Figure S3. H** and **I**. In this mode the 2-chloro-4-fluoro-phenyl ring T-stacks with Phe118 and forms favorable hydrophobic packing with Ala60. The chlorine atom of this ring formed favorable electrostatics interactions with the side-chains of Arg121 and Asn28, and the fluorine formed favorable electrostatics interactions with the side-chain of Ser65. The oxygen atom of the central methoxy formed a hydrogen bond with the side-chain of Ser82, and it formed favorable electrostatics interactions with the side-chain hydroxyl of Tyr24. The oxygen of the terminal methoxy group had favorable electrostatics interactions with the side-chain of Thr102 and with the backbone amino of Ala101. The carbonyl of the peptide bond displayed favorable electrostatics interactions with both the backbone amino and the side-chain hydroxyl of Thr62.

**Docking results against the templates**

When both the original model of DG70 and the model that was minimized with Avogadro (in which the peptide bonds were allowed to rotate during the calculations with these two ligand models) were docked against the holo version of the crystal structure of the template PDB ID: 3ou2 (DhpI, a phosphonate O-methyltransferase from *Streptomyces luridus* bound to SAH), their top-ranked modes superimposed on each other and displayed docking scores that were within 0.1 kcal/mol of each other. Their docked modes had amide bonds that were nearly trans (the expected conformation). Similar to the interactions that were observed when docking DG70 to the MenG models, this ligand displayed several base stacking interactions when it docked against 3ou2. Using the residue numbering scheme from PDB ID: 3ou2, the terminal methoxy phenyl ring was predicted to base stack with Tyr15, the central methoxy phenyl ring was predicted to T-stack with His119, and the 2-chloro-4-fluoro-phenyl ring base stacked with Tyr29 in the docked modes. The NH group in the amide bond was predicted to hydrogen bond with the hydroxyl in the side-chain of Tyr15. The carbonyl in the amide bond was predicted to form favorable electrostatic interactions with the hydroxyl in the side-chain of Tyr29. Similarly, the oxygen of the methoxy group attached to the central phenyl ring was predicted to form favorable electrostatic interactions with the hydroxyl in the side-chain of Tyr22, and the oxygen in the methoxy group attached to the terminal phenyl ring was predicted to form favorable electrostatic interactions with the backbone NH group of Gly76.

When DG70 was docked against the PDB ID: 2avn template (a ubiquinone/menaquinone biosynthesis methyltransferase-related protein from *Thermotoga maritima* MSB8), three models of DG70 (original and two different minimized outputs, which all had a rigid amide bond during the calculations) produced top-ranked modes that all superimposed on each other and had FEB docking scores (for estimated Free Energy of Binding) that were within 0.1 kcal/mol of each other (the smallest increment in FEB scores output by AutoDock Vina). This consistency in the results adds weight to the predicted binding mode. The 2-chloro-4-fluoro-phenyl ring was predicted to form base stacking interactions with Tyr9 (using the residue numbering scheme from PDB ID: 2avn). The carbonyl of the amide bond was predicted to hydrogen bond with the hydroxyl in the side-chain of Tyr16. The oxygen atoms in the two methoxy groups were predicted to form favorable electrostatic interactions with the side-chains of Thr139, Trp56, Thr53, Lys55 (if that Lys side-chain moved about an Angstrom closer), and with the backbone NH group of Gly110. With slight changes in the rotamers (i.e., in the chi1 and chi2 angles of the side-chains), the terminal methoxy phenyl ring could also form T-stacking interactions with Trp25 and/or His29.

The types of interactions that were observed in the docking results against the templates from PDB ID: 3ou2 and 2avn were also frequently observed when the six different models of DG70 were docked against many of the different original or minimized versions of the different MenG homology models and domain-threaded models. The central methoxy phenyl ring was often predicted to form T-stacking interactions with the key residue Trp75, but sometimes it instead base stacked with Tyr184. The terminal methoxy phenyl ring was often predicted to form T-stacking or base stacking interactions with the key residue Phe118.

**Additional Discussion of the Resistant Mutants**

Based on (a) the results of the sequence analyses, (b) inspecting the positions in the templates that correspond to the four key residues that mutate upon DG70 treatment, and (c) the predicted structures in the collection of different MenG models, we are confident that at least three of these four key residues are located in the active site of MenG. Phe118 is likely within the substrate binding region of the active site, with Val20 near it in three-dimensional space, but Ser188 might be closer to the edge / rim of the active site, or within the secondary shell that surrounds the active site residues. These three residues are also highly conserved in the multiple sequence alignment, which suggests that they might be important for the function, folding, and/or stability of this class of enzymes. The facts that (a) these three residues are highly conserved and (b) that the particular substitutions that arose during treatment with DG70 are very rare in the sequence alignment, both support the notion that these observed substitutions are a direct result of needing to evade DG70 treatment. Conversely, in some models Trp75 (or the residue corresponding to it in the homologs) was within the active site, but in others it was on the opposite end of the domain. Trp75 is the only one of the four residues that was not well conserved in the multiple sequence alignment of hundreds of MenG sequences.

In addition to the direct mechanism of resistance proposed for the Ser188Ala mutation (in the main text), an indirect, more complicated mechanism is also a possible contributor to DG70 resistance. Since the Arg121 side-chain interacts with both the key residue Ser188 and also with some of the predicted binding modes of DG70, an indirect effect could occur in this dynamic, flexible target. If this Arg residue is necessary for the catalytic mechanism of MenG (which would suggest that it could not be mutated without compromising the fitness of the enzyme), then perhaps the Ser188 to Ala mutation could alter the dynamic conformational preferences (to cause “gating” behavior that hinders the ability of DG70 to access the active site) and/or increase the energetic cost associated with conformational changes of this Arg121 residue (i.e., increasing an enthalpic penalty required to form a MenG:DG70 complex), thereby impeding DG70 binding or increasing its off rate. This indirect mechanism involving the removal of favorable interactions between Ser188 and Arg121, to thus prevent favorable interactions between DG70 and particular conformations of that Arg, is in line with the observation that Arg121 is highly conserved in the multiple sequence alignment (i.e., it is invariant in the closest 226 homologs to MenG).

**Additional Discussion of the Inactivity of DG70 in Non Tubercle Mycobacteria**

Of the Mycobacteria examined, only *M. tuberculosis* and BCG are susceptible to DG70 treatment, and their MenG proteins display 100% sequence identity to each other. Against the five other species of Mycobacteria tested, DG70 displayed an MIC >100 µM, and their MenG proteins had 64.5 to 88.9% sequence identity to MenG from *M. tuberculosis*. Key residues displayed variations in sequence that could help explain these observed MIC trends. *M. fortuitum* naturally has Ala at the position corresponding to Gly99 (using the *M. tuberculosis* numbering scheme); a similar substitution of Gly99Val is a DG70-resistant mutant that was selected in BCG. Similarly, *M. smegmatis, M. fortuitum,* and *M. abscessus* have Glu at the position corresponding to Asp7. The extra methylene linker in the longer side-chain of Glu could cause it to steric clash with DG70 (instead of DG70 forming a hydrogen bond with the side-chain of Asp, as was observed in the docking studies). The prediction that it is important for the amino group in the amide linker in DG70 to form a strong hydrogen bond with the carboxylate group in Asp7 is supported by the observations that (a) Asp7His was a DG70-resistant mutant selected in *M. bovis,* and (b) the N-methylated analog JSF-2949 is much less potent against *M. tuberculosis* than DG70 (39 versus 2.4 µg/ml, respectively). In some of the docking studies, the side-chains of Lys23 and Arg173 displayed favorable interactions with DG70. *M. marinum*, *M. avium*, *M. smegmatis*, *M. fortuitum*, and *M. abscessus* all have Arg instead of the much more flexible Lys23, which could impede the formation of that favorable interaction with DG70. Similarly, instead of Arg173, *M. smegmatis* and *M. fortuitum* have Thr, which is much smaller and has both polar and hydrophobic groups at the end of its short branches (rather than having a positively charged group at the end of a long, very flexible, linear side-chain), which could also prevent the formation of that favorable interaction with DG70.

**References**

1. **Lew JM, Kapopoulou A, Jones LM, Cole ST.** 2011. TubercuList--10 years after. Tuberculosis (Edinb) **91:**1-7.

2. **Drozdetskiy A, Cole C, Procter J, Barton GJ.** 2015. JPred4: a protein secondary structure prediction server. Nucleic Acids Res **43:**W389-394.

3. **Krogh A, Larsson B, von Heijne G, Sonnhammer EL.** 2001. Predicting transmembrane protein topology with a hidden Markov model: application to complete genomes. J Mol Biol **305:**567-580.

4. **Pundir S, Martin MJ, O'Donovan C, UniProt C.** 2016. UniProt Tools. Curr Protoc Bioinformatics **53:**1 29 21-15.

5. **Finn RD, Clements J, Arndt W, Miller BL, Wheeler TJ, Schreiber F, Bateman A, Eddy SR.** 2015. HMMER web server: 2015 update. Nucleic Acids Res **43:**W30-38.

6. **Finn RD, Clements J, Eddy SR.** 2011. HMMER web server: interactive sequence similarity searching. Nucleic Acids Res **39:**W29-37.

7. **Buchan DW, Minneci F, Nugent TC, Bryson K, Jones DT.** 2013. Scalable web services for the PSIPRED Protein Analysis Workbench. Nucleic Acids Res **41:**W349-357.

8. **Jones DT.** 1999. Protein secondary structure prediction based on position-specific scoring matrices. J Mol Biol **292:**195-202.

9. **Berman HM, Westbrook J, Feng Z, Gilliland G, Bhat TN, Weissig H, Shindyalov IN, Bourne PE.** 2000. The Protein Data Bank. Nucleic Acids Res **28:**235-242.

10. **Rose PW, Prlic A, Bi C, Bluhm WF, Christie CH, Dutta S, Green RK, Goodsell DS, Westbrook JD, Woo J, Young J, Zardecki C, Berman HM, Bourne PE, Burley SK.** 2015. The RCSB Protein Data Bank: views of structural biology for basic and applied research and education. Nucleic Acids Res **43:**D345-356.

11. **Altschul SF, Gish W, Miller W, Myers EW, Lipman DJ.** 1990. Basic local alignment search tool. J Mol Biol **215:**403-410.

12. **Altschul SF, Madden TL, Schaffer AA, Zhang J, Zhang Z, Miller W, Lipman DJ.** 1997. Gapped BLAST and PSI-BLAST: a new generation of protein database search programs. Nucleic Acids Res **25:**3389-3402.

13. **Biasini M, Bienert S, Waterhouse A, Arnold K, Studer G, Schmidt T, Kiefer F, Gallo Cassarino T, Bertoni M, Bordoli L, Schwede T.** 2014. SWISS-MODEL: modelling protein tertiary and quaternary structure using evolutionary information. Nucleic Acids Res **42:**W252-258.

14. **Singh S, McCoy JG, Zhang C, Bingman CA, Phillips GN, Jr., Thorson JS.** 2008. Structure and mechanism of the rebeccamycin sugar 4'-O-methyltransferase RebM. J Biol Chem **283:**22628-22636.

15. **Lee JH, Bae B, Kuemin M, Circello BT, Metcalf WW, Nair SK, van der Donk WA.** 2010. Characterization and structure of DhpI, a phosphonate O-methyltransferase involved in dehydrophos biosynthesis. Proc Natl Acad Sci U S A **107:**17557-17562.

16. **Dai YN, Zhou K, Cao DD, Jiang YL, Meng F, Chi CB, Ren YM, Chen Y, Zhou CZ.** 2014. Crystal structures and catalytic mechanism of the C-methyltransferase Coq5 provide insights into a key step of the yeast coenzyme Q synthesis pathway. Acta Crystallogr D Biol Crystallogr **70:**2085-2092.

17. **Lobley A, Sadowski MI, Jones DT.** 2009. pGenTHREADER and pDomTHREADER: new methods for improved protein fold recognition and superfamily discrimination. Bioinformatics **25:**1761-1767.

18. **McGuffin LJ, Jones DT.** 2003. Improvement of the GenTHREADER method for genomic fold recognition. Bioinformatics **19:**874-881.

19. **Jones DT.** 1999. GenTHREADER: an efficient and reliable protein fold recognition method for genomic sequences. J Mol Biol **287:**797-815.

20. **Sanner MF.** 1999. Python: a programming language for software integration and development. J Mol Graph Model **17:**57-61.
