# Supplementary material for: A Novel Small-Molecule Inhibitor of the Mycobacterium tuberculosis Demethylmenaquinone Methyltransferase MenG Is Bactericidal to Both Growing and Nutritionally Deprived Persister Cells
Source: mBio. 2017 Feb 14;8(1):e02022-16. doi: 10.1128/mBio.02022-16 (PMC5312080; doi:10.1128/mBio.02022-16)
Supplement: FIG S2 [file mbo001173186sf2.docx]

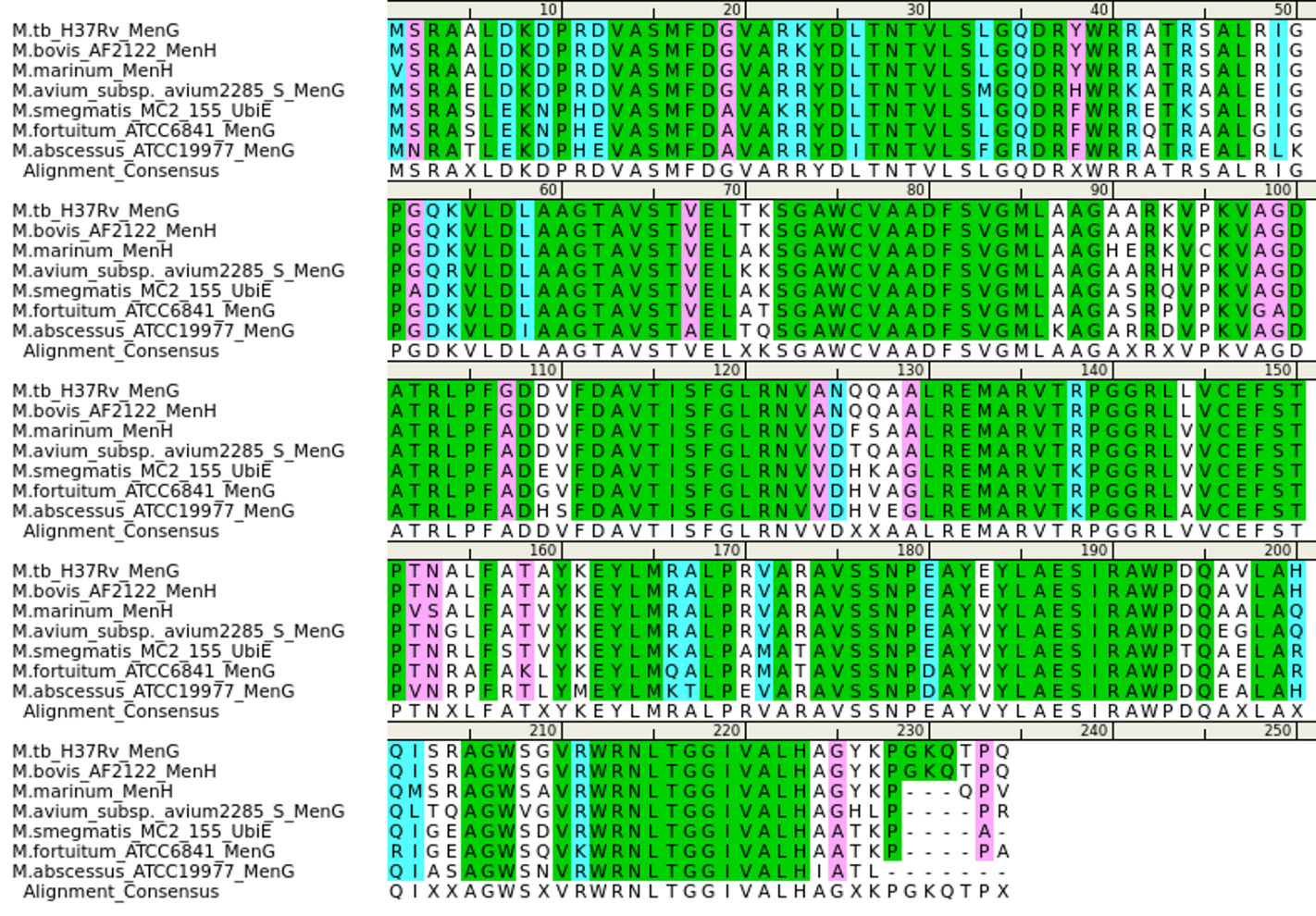


**Figure S2. Multiple sequence alignment of *Mtb* MenG versus other mycobacteria against which the activity of DG70 was tested.** A multiple sequence alignment of MenG from *Mtb*, BCG (*M.bovis*), *M.marinum*, *M.avium*, *M.smegmatis*, *M.fortuitum*, and *M.abscessus* was performed in Discovery Studio 4.5. Color legend: green = identical residues, cyan = strong conservation, magenta = weak conservation, and no highlight = non-matching. The consensus alignment was determined using a 51% sequence identity cut-off.
