# Supplementary material for: A Novel Small-Molecule Inhibitor of the Mycobacterium tuberculosis Demethylmenaquinone Methyltransferase MenG Is Bactericidal to Both Growing and Nutritionally Deprived Persister Cells
Source: mBio. 2017 Feb 14;8(1):e02022-16. doi: 10.1128/mBio.02022-16 (PMC5312080; doi:10.1128/mBio.02022-16)
Supplement: TABLE S1 [file mbo001173186st1.docx]

**Table S1: DG70 MICs in non-tuberculosis mycobacterial strains, ESKAPE pathogens and surrogate select agents.**

| **Strain** | **DG70(μg/ml)** |
| --- | --- |
| *M. tuberculosis* | 4.8 |
| *M.bovis* BCG | 2.4 |
| *M. fortuitum* | >40 |
| *M. abcessus* | >40 |
| *M. smegmatis* | >40 |
| *M. marinum* | >40 |
| *M. avium* | >40 |
| *M. fortuitum* | >40 |
| *A. baumanii* | >20 |
| *E. cloacae* | >20 |
| *E. faecium* | >20 |
| *Klebsiella pneumoniae* | >20 |
| *Pseudomonas aeruginosa* | >20 |
| *S. aureus MRSA* | >20 |
| *S. epidemis 14490* | >20 |
| *Bacillus cereus* | >40 |
| *Burkholderia cepacia* | >40 |
| *Yersinia pseudotuberculosis* | >40 |
| *Brucella neotomae* | >40 |
| *Francisella philomiragia* | >40 |
| *Legionella pneumophila* | >40 |
