# Supplementary material for: A Novel Small-Molecule Inhibitor of the Mycobacterium tuberculosis Demethylmenaquinone Methyltransferase MenG Is Bactericidal to Both Growing and Nutritionally Deprived Persister Cells
Source: mBio. 2017 Feb 14;8(1):e02022-16. doi: 10.1128/mBio.02022-16 (PMC5312080; doi:10.1128/mBio.02022-16)
Supplement: FIG S3 [file mbo001173186sf3.docx]

**Figure S3: Additional predicted binding modes of DG70 with models of MenG**. In all panels, the residues that mutate in *Mtb* upon DG70 treatment are shown as CPK with light blue carbon atoms. Residues that mutate in BCG are displayed with purple carbon atoms, and additional residues that formed strong favorable interactions with the docked mode (but that did not mutate in selection experiments) are shown with gray carbons (as either thin sticks or as CPK). In **A** and **B**, the multi-template based homology model of MenG from MODELLER that had the top DOPE score is displayed, with **B** displaying the docked binding mode of DG70 to this model, using ball-and-sticks with green carbon atoms. **C** and **D** depict the multi-template based homology model from MODELLER that had the 2^nd^ highest DOPE score; the docked mode of DG70 is displayed in **D** (with Arg121 shown as thin gray sticks). **E** and **F** show the model made by threading MenG onto the known CATH domains using pDomTHREADER in PsiPRED, and each of these panels displays a different top-ranked docked mode that was produced against this target. **G-I** display the MenG model created by threading its sequence onto the crystal structure of PDB ID: 4obx using pGenTHREADER in PsiPRED, and two different views of the same docked mode are depicted in **H** and **I**.

**
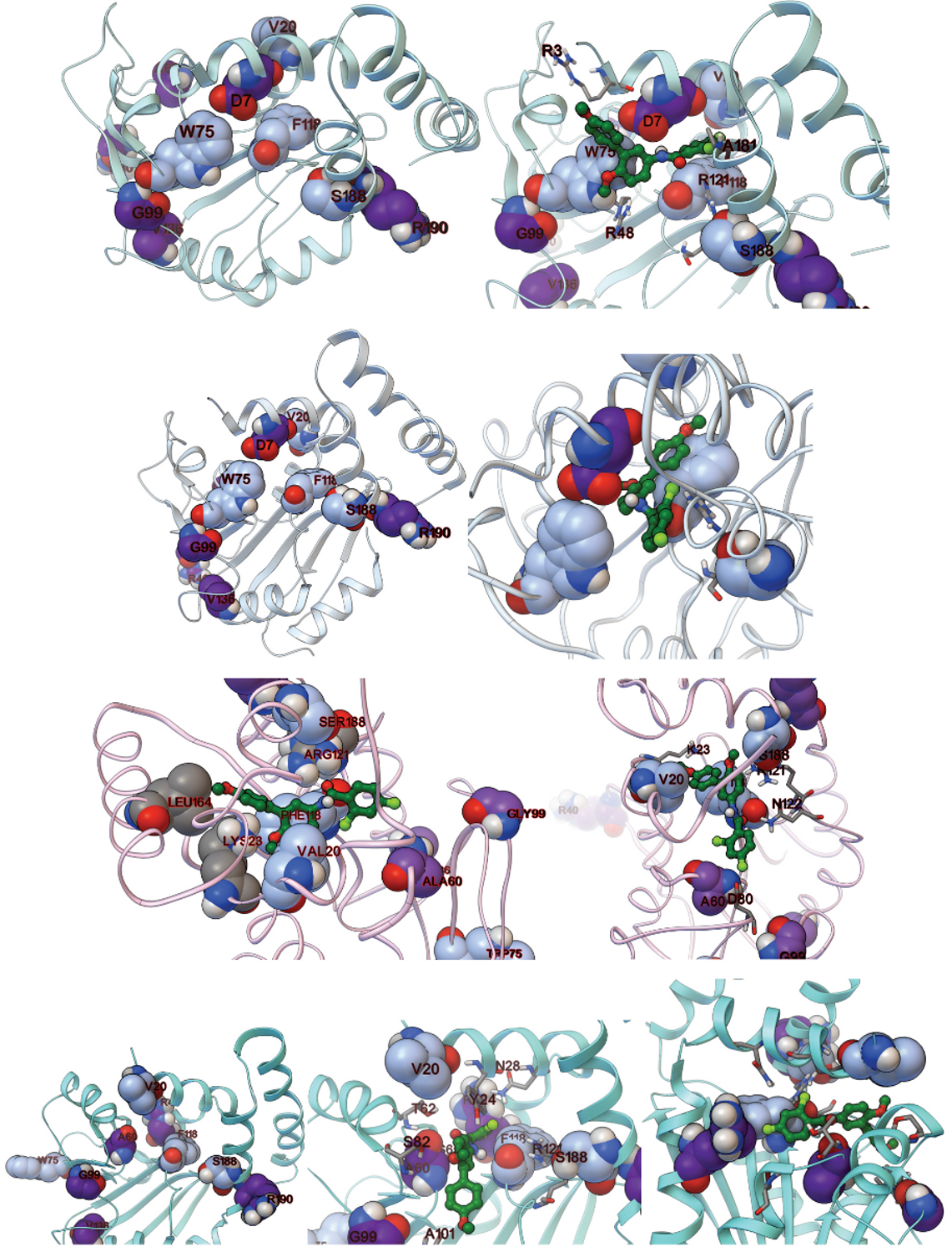
**

**I**

**H**

**G**

**F**

**E**

**A**

**B**

**C**

**D**
