# Supplementary material for: A Novel Small-Molecule Inhibitor of the Mycobacterium tuberculosis Demethylmenaquinone Methyltransferase MenG Is Bactericidal to Both Growing and Nutritionally Deprived Persister Cells
Source: mBio. 2017 Feb 14;8(1):e02022-16. doi: 10.1128/mBio.02022-16 (PMC5312080; doi:10.1128/mBio.02022-16)
Supplement: FIG S4 [file mbo001173186sf4.docx]

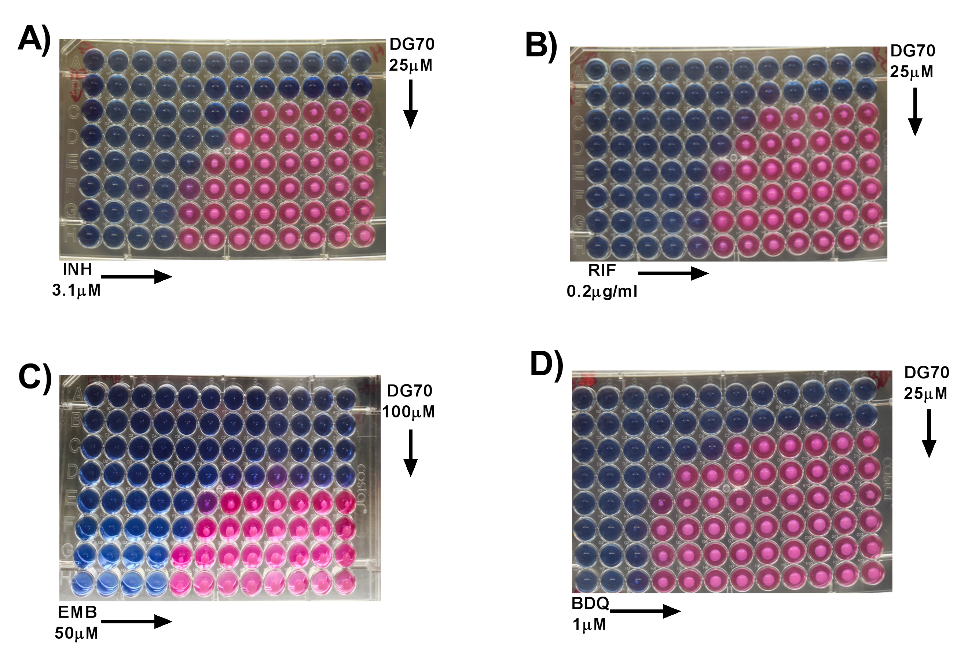


**Figure S4: Representative results from the Alamar Blue checkerboard assay for DG70 and known anti-TB drugs in *Mtb.*** *Mtb* H37Rv was treated with two-fold serial dilutions of DG70 and known anti-TB drugs A) isoniazid (INH), B) rifampicin (RIF), C) ethambutol (EMB) and D) Bedaquiline (BDQ). The viability is determined by resazurin (blue), which is converted to pink (resorufin) by viable bacteria.
