# Supplementary material for: A Novel Small-Molecule Inhibitor of the Mycobacterium tuberculosis Demethylmenaquinone Methyltransferase MenG Is Bactericidal to Both Growing and Nutritionally Deprived Persister Cells
Source: mBio. 2017 Feb 14;8(1):e02022-16. doi: 10.1128/mBio.02022-16 (PMC5312080; doi:10.1128/mBio.02022-16)
Supplement: TEXT S2 [file mbo001173186s2.docx]

***Scheme S1***

**Scheme S2**

**Schemes for synthesis of DG70 analogs. Scheme S1*:*** Synthesis of compounds **via Schotten-Baumann reaction. Scheme S2:** Synthesis of compound **JSF-2951**
