# Supplementary material for: A Novel Small-Molecule Inhibitor of the Mycobacterium tuberculosis Demethylmenaquinone Methyltransferase MenG Is Bactericidal to Both Growing and Nutritionally Deprived Persister Cells
Source: mBio. 2017 Feb 14;8(1):e02022-16. doi: 10.1128/mBio.02022-16 (PMC5312080; doi:10.1128/mBio.02022-16)
Supplement: FIG S5 [file mbo001173186sf5.docx]

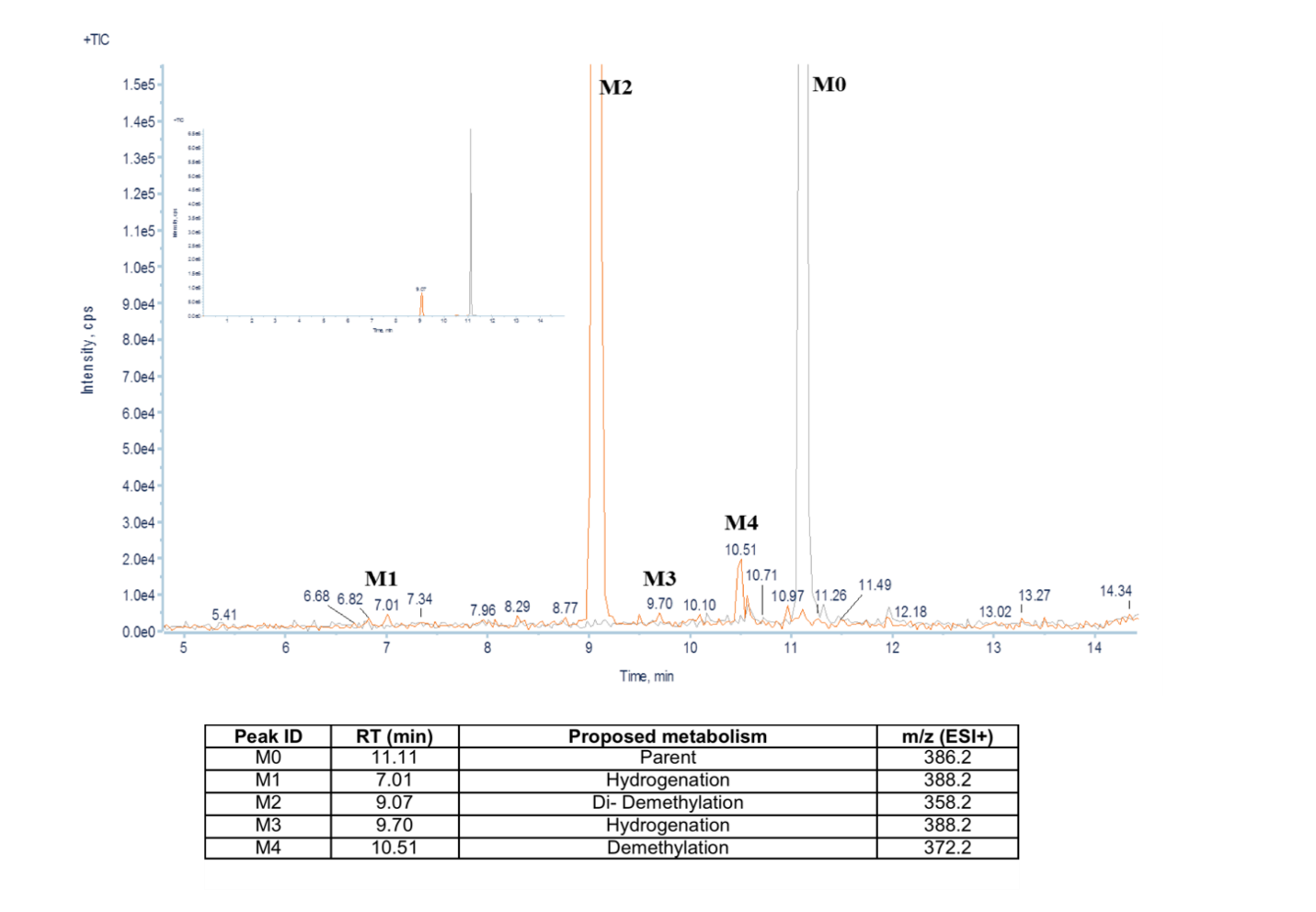


**Figure S5: Mouse Liver microsome stability of DG70.** LC-MS analysis demonstrated the major mouse liver microsome-derived metabolite of DG70 involves di-demethylation.
