# Supplementary material for: A Novel Small-Molecule Inhibitor of the Mycobacterium tuberculosis Demethylmenaquinone Methyltransferase MenG Is Bactericidal to Both Growing and Nutritionally Deprived Persister Cells
Source: mBio. 2017 Feb 14;8(1):e02022-16. doi: 10.1128/mBio.02022-16 (PMC5312080; doi:10.1128/mBio.02022-16)
Supplement: TABLE S2 [file mbo001173186st2.docx]

**Table S2: MICs of DG70 and its analogs in DG70 resistant mutants of H37Rv**

| **Name** | **MIC in *Mtb* H37Rv (μg/ml)** | **MIC in *Mtb* P3 V20A**  **(μg/ml)** | **MIC in *Mtb* P7 F118L**  **(μg/ml)** |
| --- | --- | --- | --- |
| DG70 | 2.4 | >80 | 40 |
| JSF-2912 | 9.6 | >40 | >40 |
| JSF-2908 | 4.8 | >40 | >40 |
| JSF-2909 | >77 | >80 | >80 |
| JSF-2951 | 77 | >80 | >80 |
| JSF-2949 | 39.5 | >40 | >40 |
| JSF-2950 | 39.5 | >40 | >40 |
| JSF-2910 | 9.6 | >40 | >40 |
| JSF-2907 | >77 | >80 | >80 |
| JSF-2971 | 9.6 | 40 | 40 |
| JSF-2926 | 9.6 | 40 | 40 |
